# Diversity of Metabolically Active *Bacteria* in Water-Flooded High-Temperature Heavy Oil Reservoir

**DOI:** 10.3389/fmicb.2017.00707

**Published:** 2017-04-25

**Authors:** Tamara N. Nazina, Natalya M. Shestakova, Ekaterina M. Semenova, Alena V. Korshunova, Nadezda K. Kostrukova, Tatiana P. Tourova, Liu Min, Qingxian Feng, Andrey B. Poltaraus

**Affiliations:** ^1^Laboratory of Petroleum Microbiology, Research Center of Biotechnology, Winogradsky Institute of Microbiology, Russian Academy of SciencesMoscow, Russia; ^2^Dagang Oil Field Group Ltd.Tianjin, China; ^3^Engelhardt Institute of Molecular Biology, Russian Academy of SciencesMoscow, Russia

**Keywords:** petroleum reservoir, 16S rRNA gene clone library, cDNA of 16S rRNA, metabolic activity, gene *alkB*, near-bottom zone of injection well, biogeochemical processes and cycles

## Abstract

The goal of this work was to study the overall genomic diversity of microorganisms of the Dagang high-temperature oilfield (PRC) and to characterize the metabolically active fraction of these populations. At this water-flooded oilfield, the microbial community of formation water from the near-bottom zone of an injection well where the most active microbial processes of oil degradation occur was investigated using molecular, cultural, radiotracer, and physicochemical techniques. The samples of microbial DNA and RNA from back-flushed water were used to obtain the clone libraries for the 16S rRNA gene and cDNA of 16S rRNA, respectively. The DNA-derived clone libraries were found to contain bacterial and archaeal 16S rRNA genes and the *alk*B genes encoding alkane monooxygenases similar to those encoded by *alkB*-*geo1* and *alkB*-*geo6* of geobacilli. The 16S rRNA genes of methanogens (*Methanomethylovorans, Methanoculleus, Methanolinea, Methanothrix*, and *Methanocalculus*) were predominant in the DNA-derived library of *Archaea* cloned sequences; among the bacterial sequences, the 16S rRNA genes of members of the genus *Geobacillus* were the most numerous. The RNA-derived library contained only bacterial cDNA of the 16S rRNA sequences belonging to metabolically active aerobic organotrophic bacteria (*Tepidimonas, Pseudomonas, Acinetobacter*), as well as of denitrifying (*Azoarcus, Tepidiphilus, Calditerrivibrio*), fermenting (*Bellilinea*), iron-reducing (*Geobacter*), and sulfate- and sulfur-reducing bacteria (*Desulfomicrobium, Desulfuromonas*). The presence of the microorganisms of the main functional groups revealed by molecular techniques was confirmed by the results of cultural, radioisotope, and geochemical research. Functioning of the mesophilic and thermophilic branches was shown for the microbial food chain of the near-bottom zone of the injection well, which included the microorganisms of the carbon, sulfur, iron, and nitrogen cycles.

## Introduction

Microbial communities of petroleum reservoirs have been extensively studied using molecular, radioisotope, and culture-based techniques (Belyaev et al., 1982; Nazina et al., 1985, 1995; Voordouw et al., 1996; Magot et al., 2000; Bonch-Osmolovskaya et al., 2003; Orphan et al., 2003). While the results of molecular and culture-based studies of a number of environments exhibited considerable differences, molecular research on oilfield microorganisms revealed no significant differences from the results of culture-based studies in respect of diversity of the functional components of the community (Nazina et al., 1985, 2006; Voordouw et al., 1996; Youssef et al., 2009a).

Anaerobic microorganisms reducing sulfate, thiosulfate, elemental sulfur, and Fe^3+^, fermentative and syntrophic bacteria, acetogens, and methanogens were isolated from oilfields (Stetter et al., 1993; Magot et al., 2000; Takahata et al., 2000; Bonch-Osmolovskaya et al., 2003; Orphan et al., 2003). Aerobic bacteria detected in these environments are considered contaminants arriving into the reservoir from the surface during drilling or with injection water. Analysis of 12 metagenomes from hydrocarbon resource environments (An et al., 2013) revealed that although anaerobic communities were common, cores from oil sands and coal beds had unexpectedly high proportions of aerobic hydrocarbon-degrading bacteria. Moreover, most metagenomes had high proportions of genes for the enzymes involved in aerobic hydrocarbon metabolism. Head et al. (2014) evaluated several hypotheses that might explain the occurrence of organisms conventionally considered to be aerobic, in nominally anoxic petroleum reservoir habitats. These hypotheses are: “(1) Samples were exposed to oxygen during sampling, transport and storage allowing growth of aerobic organisms initially present at low abundance. (2) Oxygen was supplied externally to the reservoir by meteoric water. (3) A ‘cryptic’ aerobic community that uses *in situ* generated oxygen may be present in some petroleum systems. (4) The aerobes detected are in fact capable of anaerobic metabolism.”

Microbial diversity in formation water from production wells and in the water from the oil separation system injected into the reservoir was studied using analysis of the 16S rRNA clone libraries and pyrosequencing of the sequences obtained from total DNA (Duncan et al., 2009; Gieg et al., 2010; Van der Kraan et al., 2010; Ren et al., 2011; Gao et al., 2015). In injected water, the composition of the microbial community depended on the water source (seawater, surface fresh water, or produced water injected back into the reservoir) and inherited, respectively, marine communities, freshwater ones, or those similar to the reservoir communities. In some cases the similarity between communities of injected water and of formation water from production wells was low, indicating the specificity of the oilfield microbial community (Ren et al., 2011; Gao et al., 2015). Microorganisms best adapted to the conditions established in the reservoir predominate in the community, unless no selective pressure is applied by addition of exogenous electron donors or acceptors (e.g., of nitrate and molasses, respectively).

Investigation of oilfield microorganisms by stopping the operation of an injecting well and switching it into the back flow regime was initiated by Russian researchers in the 1960s (Kuznetsova and Li, 1964). Contour water-flooding in order to maintain reservoir pressure was shown to result in injection of microorganisms and dissolved oxygen, thus creating favorable conditions for oil oxidation in the near-bottom zone of injection wells. The microorganisms of the near-bottom zone of injection wells in the terrigenous oilfields of Tatarstan, Western Siberia, and Kazakhstan were studied by culture-based and radioisotope techniques. The water flowing from the near-bottom zone was shown to contain high numbers of aerobic and anaerobic microorganisms belonging to diverse physiological groups, as well as the highest incorporation of ^14^C-labeled octadecane into microbial biomass and high rates of sulfate reduction and methanogenesis compared to those of formation water from production wells (Belyaev et al., 1982; Rozanova and Nazina, 1982; Nazina et al., 1985, 1995; Rozanova et al., 1995).

Electron microscopy, analysis of the fatty acid profile, and incorporation of ^14^C-labeled acetate and glucose in the microbial biomass were used to study the microbial community of the near-bottom zone of injection well in a Californian petroleum reservoir (McKinley et al., 1988). The results indicated the presence of anaerobic and facultative anaerobic prokaryotes in the community, which contained a considerable share of gram-negative bacteria. Molecular techniques were used to investigate the composition of microbial communities in the near-bottom zone of injection wells of the North Sea and Chinese petroleum reservoirs (Bødtker et al., 2009; Gao et al., 2015). Water samples from nitrate-treated reservoir contained lower numbers of sulfate-reducing bacteria and were enriched with denitrifying bacteria (Bødtker et al., 2009). The information on microbial abundance, biodiversity, and activity in the zone of inflow of the surface water into the reservoir, which is characterized by fluctuating physicochemical conditions, is scarce.

Analysis of the libraries of the cDNA of 16S rRNA fragments obtained using ribosomal RNA has been used for description of microbial communities (Mills et al., 2005, 2012; Moeseneder et al., 2005). This method is based on the fact that a microbial cell contains more ribosomes than ribosomal operons (16S rRNA genes). Bacterial and archaeal cells are known to contain 1–15 and 1–5 ribosomal operons (Acinas et al., 2004; Stoddard et al., 2015). Since the number of ribosomes and the ribosomal RNA content increase in actively growing cells, analysis of ribosomal RNA is used to reveal the metabolically active cell forms (Moeseneder et al., 2005). Using ribosomal RNA as an indicator of microbial activity in environmental communities is known to have its limitations. Some of them were discussed in the review by Blazewicz et al. (2013). It was shown that “concentration of rRNA and growth rate is not always simply correlated; the relationship between rRNA concentration and growth rate can differ significantly among taxa; and dormant cells can contain high numbers of ribosomes.” However, although the statements concerning prokaryotic metabolic activity derived from analysis of the RNA-based libraries may be open to doubt, comparison of the DNA- and RNA-based libraries provides for more complete characterization of microorganisms in environmental communities.

Trials of technologies for microbial enhancement of oil recovery (MEOR) were carried out at the Kongdian bed of the Dagang oilfield and resulted in additional recovery of over 36,000 t of oil (Nazina et al., 2006, 2008). Over 40 parameters of formation water, oil, and gas from production wells were monitored during the trial. Microbial diversity in formation water and enrichment cultures was studied by analysis of the 16S rRNA gene and of the *alkB* functional gene encoding alkane monooxygenase, the key enzyme of *n*-alkane aerobic degradation (Nazina et al., 2006; Shestakova et al., 2011a,b). The functional activity and phylogenetic diversity of metabolically active prokaryotes in the near-bottom zone of injection wells, where the processes of oil biodegradation are the most intensive, remain poorly studied.

The goal of the present work was to investigate the composition of the microbial community in formation water from the near-bottom zone of an injection well in a high-temperature Dagang olifield (China) using comparative analysis of the DNA- and RNA-derived libraries of the 16S rRNA genes and cDNA of 16S rRNA and of the *alkB* functional gene and to compare these results with the data of cultural, physicochemical, radioisotope, and stable isotope research.

## Materials and methods

### Site and sample description

Formation water from the Kongdian bed of the Dagang oilfield (Hebei Province, China) was studied in the present work. The studied sandstone oil-bearing horizons of the Kongdian bed are located at the depths of 1,206−1,435 m below sea level and have a temperature of 59°C; the average porosity is 33%, and the stratum permeability is 1.878 μm^2^. Kongdian oil had a density of about 0.9459−0.9549 g/cm^3^. The detailed oil composition is given by Jiménez et al. (2012). The original formation water of hydrocarbonate–sodium type had a total salinity of 5,612 mg l^−1^, pH 7.1–7.6. The oil gas contained methane (95–98%), higher C_2_–C_5_ homologs (0.8–1.8%), N_2_ (0.5–3.3%), and CO_2_ (0.06–0.77%). Detailed characteristics of the bed are presented earlier (Nazina et al., 2006, 2008).

The Dagang oilfield has been exploited for about 30 years with water flooding in order to maintain the stratal pressure. The co-produced formation waters (separated from oil) of this oilfield were used for injection. As a result, the chemical composition of the injection water was similar to that of the formation water. The process of oil and water separation usually takes 2 or 3 h. The water becomes enriched with dissolved oxygen (0.8–2.6 mg O_2_ per liter); its temperature drops from 59° to 30–50°C. Then it is injected into the oil stratum through the injection wells. Within the oil field complex, several production wells are serviced by one injection well during water-flooding, with the distances to the respective injection well ranging from 50 to 300 m. The hydrodynamic links between the injection and production wells, as well as the direction of the hydrodynamic flows of injection water at the North block were reported earlier (Nazina et al., 2007a,b). Hydraulic residence times for injected waters are typically on the order of 40–80 days. All injection wells at the North block of the Kongdian bed operated in a stable regime during the period of observations in 2000–2007. Injection of an oxygen source and nitrogen and phosphorous salts was performed into all injection wells at the same time. The layout of the injection and production wells at the Kongdian bed is given on Figure S1.

In 2001–2003, the water–air mixture with diammonium phosphate was pumped into the injection wells every month during the period from March to September (a total of 15 injection cycles). In 2004, five cycles of injection (during the period from September to December 2004), and in 2005, 10 cycles of injection of diammonium phosphate with a water–air mixture (June through December 2005) were carried out. During the period from April to December 2006, 10 cycles of technological treatment were carried out; among them 3 cycles were similar to those of 2004–2005, and 7 cycles of the treatment included injection of mineral salts and H_2_O_2_ solution as a source of oxygen instead of the water-air mixture. All the injection wells of the North block of the Kongdian bed were involved in the experiment (Figure S1, Nazina et al., 2007b).

Formation water from injection well 1098, which was at the time in the back-flow regime mode, was collected prior to the trial of the biotechnology for enhanced oil recovery and at several points during the trial. Formation water flowed out under the oilfield pressure and was collected at the well head of the injection well. The outflowing volume in the long-operated Kogdian bed was low (~30 m^3^). Water samples from the near-bottom zone of the injection well (8 m^3^) were used for comparative analysis. It differed in the composition of water, which contained numerous mucous particles, oil, and black sediment. Samples of injection water (collected prior to injection) and of the water from production wells were also studied. Formation fluid was collected into sterile 0.5-L bottles filled to capacity and used for microbiological and radioisotope analyses within a day. The sample of formation water (1 L) used for the isolation of nucleic acids was collected in December 2006 and fixed with ethanol (1:1) at the time of sampling. In the laboratory, water was separated from oil by adding Triton X100 to 0.1% and washing with hexane. Prior to molecular analyses, fixed water was stored at 4°C.

### Enumeration of microorganisms

The number of cultivable microorganisms belonging to the major metabolic groups was determined by inoculating 10-fold dilutions of formation water samples into liquid media (in duplicates or in triplicate). The results were calculated using the McCready tables of the most probable number. The number of hydrocarbon-oxidizing bacteria was determined using a mineral medium supplemented with a mixture of C_10_–C_22_ *n*-alkanes (2% vol/vol). Anaerobic organotrophic bacteria with fermentative metabolism were enumerated on the medium supplemented with peptone (4 g l^−1^) and glucose (10 g l^−1^). The numbers of sulfate-reducing bacteria were determined by the increase in hydrogen sulfide in the dilution series in Postgate B medium with sodium lactate (4 g l^−1^) supplemented with microelements and reduced with 200 mg l^−1^ Na_2_S · 9H_2_O. The numbers of methanogens were assayed by the methane increase in the dilution series in the Zeikus's media with acetate (2 g l^−1^) or H_2_ plus CO_2_ (80:20; atmospheric pressure), supplemented with microelements and yeast extract (1 g l^−1^) and reduced with 500 mg l^−1^ Na_2_S · 9H_2_O (references to the description of the media were cited in a publication Bonch-Osmolovskaya et al., 2003). The inoculated media were incubated for 30 days. Thermophilic bacteria were enumerated at 60°C; mesophilic bacteria, at 30°C. All cultures were then examined using an Olympus phase contrast microscope. Microbial metabolites in the medium (H_2_S, CH_4_, CO_2_, H_2_) and the chemical composition of formation water were analyzed as described previously (Bonch-Osmolovskaya et al., 2003).

### Radioisotope methods and stable carbon isotope analysis

The rates of sulfate reduction and lithotrophic and aceticlastic methanogenesis were determined by radioisotope techniques using the labeled compounds Na_2_^35^SO_4_, NaH^14^CO_3_, and ^14^CH_3_COONa, respectively, as described previously (Bonch-Osmolovskaya et al., 2003). To measure the rates of thermophilic and mesophilic processes, aliquots of formation water were incubated with labeled substrates at 60° and 30°C, respectively. The stable isotope composition of mineral carbonates dissolved in the formation waters was analyzed by Craig's method in a Thermo Finnigan “Delta V Advantage Plus” mass spectrometer (Thermo Finnigan, United States) with accuracy of ±0.5‰ (Craig, 1953).

### Construction and analysis of 16S rRNA gene and cDNA of 16s rRNA clone libraries

#### DNA and RNA extraction and reverse transcription-PCR

Formation water was preliminary filtered through 1.2-μm GF/C filter (Whatman Glass Microfilters, United States), and the biomass from 1 L was concentrated by filtration through 0.22-μm membranes (Millipore, United States). Total DNA was isolated from formation water using the “DNeasy Blood and Tissue Kit” (QIAGEN, Germany) according to the manufacturer's recommendations. The purified DNA preparation was dissolved in 100 μl Milli-Q water and used as a template for PCR. Attempt at DNA isolation from 1.2-μm GF/C filters or from oil was unsuccessful. All stages of DNA and RNA isolation and the reagents used were tested in a preliminary study of an enrichment culture of hydrocarbon-oxidizing bacteria isolated from the Kongdian bed (Shestakova et al., 2011b).

Total RNA was isolated using TRIzol (TRIzol Reagent, Invitrogen, United States) according to the manufacturer's protocol. The biomass from 1 L of formation water concentrated on the 0.22-μm membrane filter (Millipore, United States) was washed off with 1 mL TRIzol and incubated for 5 min at room temperature. Then, 0.2 ml of chloroform was added, and the cell debris was precipitated by centrifugation (12,000 g) at 2–8°C for 15 min (Shestakova et al., 2011b). RNA from the water phase was precipitated with isopropanol; the sediment was washed with 75% ethanol and dried at room temperature. Control of the presence of amplifiable DNA left in the RNA extract was carried out. The isolated DNA was treated with DNAse, and PCR was performed with the universal bacterial primers in order to determine the presence of the 16S rRNA genes. The PCR product obtained was analyzed by gel electrophoresis. The result was negative, indicating the absence of DNA admixtures in the RNA preparation.

RNA was dissolved in water and treated with RNase-free RQ1 DNase (Promega, United States). The reaction mixture (10 μl) contained the following: 1× buffer (40 mM Tris–HCl, pH 8.0; 10 mM MgSO_4_; and 1 mM CaCl_2_), 2–5 μg of RNA, and 2 U of enzyme. The reaction mixture was incubated at 37°C for 30 min; the reaction was terminated by heating at 65°C. For reverse transcription, RNA was precipitated with 70% ethanol and 0.3 M sodium acetate. The total RNA preparations were stored at −70°C.

During all stages of the experiments with RNA and cDNA, plastic labware and pipettes pretreated with diethylpyrocarbonate (DEPC) were used; the solutions were also treated with 0.05% DEPC overnight at room temperature and then autoclaved at 0.5 atm for 30 min. All working surfaces were also pretreated with an antiRNase solution (AmBion, United States).

The obtained preparations of genomic DNA and total RNA were assayed by electrophoresis in 0.8–1.5% agarose gel. The nucleic acid concentration was determined on an ND-1000 spectrophotometer (NanoDrop, United States).

The first cDNA chain was synthesized using the Sileks kit for reverse transcription (Sileks, Moscow, Russia). The reaction mixture (25 μl) contained the following: 2–5 μg of total RNA, 15–20 nmol of hexanucleotide primers, 1× buffer (70 mM Tris–HCl, pH 8.3; 16.6 mM (NH_4_)_2_SO_4_, and 7.5 mM MgCl_2_), 6 mmol of dNTPs, and 100 U of M-MLV reverse transcriptase. The primers were preannealed with total RNA at 70°C for 5 min. Then, buffer, dNTPs, and the M-MLV reverse transcriptase were added; the resultant mixture was incubated at 25°C for 10 min and at 42°C for 60 min. The reaction was terminated by heating at 70°C for 10 min, and then the mixture was cooled on ice. The resultant cDNA of 16S rRNA was used immediately as a template for PCR with universal primers.

### 16S rRNA gene, cDNA, and *alkB* gene amplification

The 16S rRNA gene fragments and cDNA of 16S rRNA of representatives of the domain *Bacteria* were amplified using the universal primers 8-27f (5′-AGAGTTTGATCCTGGCTCAG-3′) and 519r (5′-G(T/A)ATTAC-CGCGGC(T/G)GCTG-3′) (Weisburg et al., 1991; Brunk et al., 1996). There were 30 PCR cycles of 94°C for 0.5 min, 50°C for 0.5 min, and 72°C for 0.5 min. The 16S rRNA genes of *Planctomycetes* were amplified using the Pla46f primer developed by Neef et al. (1998) in combination with the 519r, 1390r, and 1492r universal primers as recommended by Schmid et al. (2000). The 16S rRNA gene fragments of archaea were amplified 35 PCR cycles using the primers A109F (5′-ACG/TGCTCAGTAACACGT-3′) and A1041r (5′-GGCCATGCACCWCCTCTC-3′) (Kolganova et al., 2002). Analysis of the obtained DNA fragments was carried out by electrophoresis in 1% agarose gel stained with ethidium bromide. The PCR products were purified by DNA precipitation with a mixture of ethanol and 0.75 M ammonium acetate at room temperature.

PCR amplification of *alkB* genes was performed using degenerate oligonucleotide primers: the forward Alk-BFB primer (5′-GGT ACG GSC AYT TCT ACR TCG A-3′) and the reverse Alk-BRB primer (5′-CGG RTT CGC GTG RTG RT-3′) (Tourova et al., 2008). The reaction mixture contained the following: 50–80 ng of DNA; 5 pmol of each primer; dNTPs, 200 μM each; 0.5 U of the thermostable Taq DNA-polymerase; and 1× Taq buffer (10 mM Tris–HCl, pH 8.3; 50 mM KCl; and 2 mM MgCl_2_). Amplification was performed in a Mastercycler (Eppendorf, Germany). The PCR cycle parameters were as follows: polymerase activation (94°C for 3 min); then, 35 cycles of DNA denaturation (94°C for 0.5 min), primer annealing (60°C for 1 min), and extension (72°C for 0.5 min); and final extension (72°C for 8 min).

#### Cloning of 16S rRNA genes, cDNA of 16S rRNA fragments and alkB genes and sequence analysis

The amplified *alkB*, 16S rRNA gene and cDNA fragments of 16S rRNA (about 500 bp) were cloned into the plasmid vector pTZ57RT (Fermentas, Lithuania). Clones containing DNA inserts of the expected size (~500 bp) were sequenced using the plasmid primers M13D and M13R. Sequencing was performed on a 3,730 DNA Analyzer sequencer using a BigDye Terminator v3.1 Cycle Sequencing Kit (Applied Biosystems, United States).

The sequences of the 16S rRNA and *alkB* gene fragments were homology-searched using the BLAST software and the NCBI GenBank database (http://www.ncbi.nlm.nih.gov). The sequences were edited using the BioEdit software package (http://jwbrown.mbio.ncsu.edu/BioEdit/bioedit.html). The sequence data were aligned using the CLUSTALW v.1.75 software package (Thompson et al., 1994), with clones having similarities of 97% or above grouped into operational taxonomic units (OTUs) or phylotypes. Detection of the chimeras and checking for the secondary structure anomalies was carried out using the CHIMERA-CHECK program from the Ribosomal Database Project. Six sequences identified as possible chimeras were found. Unrooted phylogenetic trees were constructed using the methods implicated in the TREECONW software package [http://bioc-www.uia.ac.be/u/yvdp/treeconw.html] and the RDP (Ribosomal Database Project) reference sequences (http://rdp.cme.msu.edu).

### Statistical analysis

Rarefaction analysis (Heck et al., 1975) and coverage (Good, 1953) were applied to assess the representation of the phylotypes and to characterize the diversity of 16S rRNA genes of bacteria and archaea in the clone libraries generated from DNA of formation water sample and diversity of 16S cDNA transcripts of bacteria in the clone library generated from RNA of formation water. The rarefaction curves were produced with the software ANALYTIC RAREFACTION 1.3, which is available online at http://www.uga.edu/_strata/software/index.html. The coverage of the clone libraries was calculated from the equation:

C = [1 − (n_1_/N)] × 100, described by Good, where C is the homologous coverage, n_1_ is the number of phylotypes with one representative and N is the total number of clones analyzed.

The diversity and species richness of microorganisms in the libraries were analyzed by calculating the Shannon-Weaver and Simpson diversity indices and species richness estimators Chao 1 with the EstimateS program (version 8, R.K. Colwell, http://purl.oclc.org/estimates). The dominance index was calculated as the ratio between the number of clones of the predominant phylotype and the total number of clones.

### Nucleotide sequence accession numbers

The obtained nucleotide sequences were deposited in the GenBank under the following accession numbers: 16S rRNA gene fragments KY273929–KY273938, and KY273969–KY274001; cDNA of 16S rRNA transcripts KY273939–KY273965; and *alkB* genes KY273966–KY273968.

## Results

### Physicochemical and microbiological characteristics of the backflushed water from the near-bottom zone of injection well and of water from production wells of the kongdian bed

In 2001–2007, biotechnology for enhancement of oil recovery based on the activation of the stratal microorganisms was tested in the high-temperature horizons of the Kongdian bed of the Dagang oilfield (Nazina et al., 2006, 2008). This biotechnology consists in the pumping of a water–air mixture and nitrogen and phosphorus mineral salts into the oil stratum through injection wells in order to stimulate the activity of the stratal microflora, which produce oil-releasing metabolites. In 2006, hydrogen peroxide solution was used instead of the water-air mixture.

Since the main processes of oil oxidation occurred in the zone of dissolved oxygen inflow, the near-bottom zone of injection well 1098 was studied. In the sample of back-flushed water (8 m^3^) released from well 1098, diversity of thermophilic and mesophilic prokaryotes and the rates of terminal microbial processes of oil biodegradation were determined. Production wells 1002-1, 1008-1, and 1032-1, which located in the zone affected by other injection wells, were studied for the demonstration of the processes occurring in the anoxic zone of the oilfield.

Aerobic and anaerobic microorganisms were brought into the bed with injected water (Table 1). Oxygen dissolved in injected water stimulated growth of aerobic bacteria responsible for oil biodegradation and therefore also growth of the anaerobes consuming the products of oil oxidation. Injection of cooled water resulted in the propagation of both thermophilic and mesophilic microorganisms in the near-bottom zone of injection wells. This was confirmed by detection of thermophilic and mesophilic sulfate reduction and methanogenesis (Figures S2A,B). Abundance of cultivable mesophilic and thermophilic microorganisms belonging to the major physiological groups (aerobic organotrophic, anaerobic fermenting, sulfate-reducing, and methanogenic prokaryotes) in the water from the near-bottom zone of an injection well 1098 was considerably higher than in the water from production well 1032-1 (Table 1, Figure S2A). While viable mesophilic prokaryotes were detected in the water from production wells, the rates of mesophilic sulfate reduction and methanogenesis were low or zero (Figure S2C). The biotechnological treatment resulted in increased abundance of thermophilic sulfate-reducing and methanogenic microorganisms in the zone of production wells with close hydrodynamic connection to injection wells (e.g., wells 1002-1 and 1008-1, Table 1). In December 2006 the rates of anaerobic processes in the near-bottom zone of injection well 1098 and in a number of producing wells were low, probably due to H_2_O_2_ injection into the bed.

**Table 1:** **Chemical composition, numbers of thermophilic prokaryotes and rates of thermophilic sulfate-reduction and methanogenesis in the back-flushed water from the near-bottom zone of injection well 1098 (sample 8 m^3^) and in water from production wells of the Kongdian bed**.

**Parameter**	**Injection water**		**Back-flushed water from the near-bottom zone of injection well 1098, sample 8 m**^**3**^					**Production well 1002-1**			**Production well 1008-1**		**Production well 1032-1**
	**Month, year of sampling**												
	**Dec., 2000**	**May, 2006**	**Dec., 2000**	**June, 2002**	**Dec., 2005**	**May, 2006**	**Dec., 2006^b^**	**June, 2002**	**Dec., 2005**	**Dec., 2006**	**June, 2002**	**Dec., 2006**	**June, 2002**
**CONCENTRATION IN WATER, mg l^−1^**													
K^+^+Na^+^	2,283	2,185	2,272	2,286	2,400	2,172	n.m.	2,469	2,343	n.m.	2,395	n.m.	2,166
Mg^2+^	31	27	33	37	34	40	n.m.	30	36	n.m.	27	n.m.	31
Ca^2+^	59	65	52	52	64	50	n.m.	44	39	n.m.	39	n.m.	47
Cl^−^	3,414	3,193	3,396	3,396	3,440	3,193	n.m.	3,537	3,413	n.m.	3,396	n.m.	3,168
${\text{HCO}}_{3}^{-}$+${\text{CO}}_{3}^{2-}$	517	638	503	534	594	503	470	747	532	722	747	772	579
${\text{SO}}_{4}^{2-}$	0	0	0	12	135	24	91	0	24	0	12	0	12
Total salinity, mg l^−1^	6,304	6,108	6,256	6,317	6,667	5,982	n.m.	6,827	6,387	n.m.	6,616 n.m.	6,003	
Acetate/iso-butyrate, mg l^−1^	0	1.6	3.0	16.0	0.6	1.4	2.4	74.4/98.2	1.4	1.4	160.7/52.2	2.0	7.4/17.8
**NUMBER OF MICROORGANISMS, CELLS ml^−1^**													
Aerobic organotrophs	10^2^	2.5 × 10^2^	10	2.5 × 10^3^	10^4^	<10	10^4^	2.5 × 10^2^	10	10	2.5 × 10^5^	10	2.5 × 10
Fermentative	10^4^	2.5 × 10^4^	10^5^	10^7^	10^3^	2.5 × 10^4^	10^2^	≥10^7^	10	10^5^	≥10^7^	10	≥10^4^
Sulfate-reducing	10^4^	0	10^4^	2.5 × 10^5^	10^4^	2.5 × 10^5^	≥10^5^	2.5 × 10^2^	10	≥10^5^	2.5 × 10^2^	≥10^5^	<10
Methanogens (H_2_+CO_2_)	10^3^	2.5 × 10^3^	10^3^	2.5 × 10^6^	10^2^	2.5 × 10^2^	10^4^	2.5 × 10^4^	10^3^	10^4^	2.5 × 10^3^	10^4^	2.5 × 10^3^
Methanogens (Acetate)	<10	2.5 × 10^3^	10	2.5 × 10^5^	10^3^	2.5 × 10	10^3^	2.5 × 10^3^	10	10^4^	2.5 × 10^2^	10	2.5 × 10
**SULFATE REDUCTION RATE, μg S^2−^ l^−1^ day^−1^**													
	n.m.	n.m.	2.85	291.30	2,266.61	20.79	0.20	0.01	8.50	0.02	215.63	0	0.044
**METHANOGENESIS RATE, μg CH_4_ l^−1^ day^−1^**													
From NaH^14^${\text{CO}}_{3}^{-}$	n.m.n	n.m.	1.50	0.69	41.13	0.46	0.03	0.15	0.100	0.01	0.60	0.04	0
From 2-^14^C-acetate	.m.	n.m.	14.73	228.47	1.81	0.01	0.002	20.55	0.04	0.02	39.68	0.07	0.640
δ ^13^C/S(CO_2_ + ${\text{HCO}}_{3}^{-}+$ ${\text{CO}}_{3}^{2-}$), °/_*oo*_ PDB	+1.1	−3.3	+4.2^*a*^	−0.4	−8.5	−0.4	+9.9	+5.2	−2.2	+4.8	N.d.	−4.9	+6.9
Biotechnological treatment	No	H_2_O_2_	No	Air	Air	H_2_O_2_	H_2_O_2_						

*n.m., not measured*.

a *Result for water sample 1098-25 m^3^*.

b *The water sample was used for molecular studies*.

At the investigated site, formation water of the hydrocarbonate-sodium type had a low salinity (5.9–6.8 g l^−1^) and slightly alkaline reaction (pH 7.1–7.6). In the original formation water, no sulfates were present; in the course of biotechnological treatment their concentration in the near-bottom zone reaches 135 mg l^−1^ (Table 1). Sulfide (below 2 mg l^−1^) was found in the water of the near-bottom zone, but not in the fluids from production wells.

Injection of the electron acceptor (O_2_) into the bed resulted in biodegradation of oil in the near-bottom zone of injection well 1098, which was confirmed by elevated concentrations of mineral carbonates (${\text{HCO}}_{3}^{-}$ + ${\text{CO}}_{3}^{2-}$) and volatile fatty acids (VFA) in water (Table 1, Figures S3A–C). VFA concentration in formation water peaked in June 2002, after the first stage of injection of the water–air mixture with diammonium phosphate in 2001. In 2004-2006, the concentration of volatile fatty acids in formation water was considerably lower than at the onset of the field experiment, which was probably due to the oxidation of a considerable share of easily utilized components of residual heavy oil in the near-bottom zone at the initial stage of the biotechnological treatment. In the course of oil biodegradation during the treatment, isotopically light mineral carbonates were revealed, with δ^13^C values [δ^13^C/∑(CO_2_ + ${\text{HCO}}_{3}^{-}$ + CO_3_2−)] sometimes decreasing from 4.2‰ to −0.4…−8.5‰ PDB (Table 1).

Volatile fatty acids and carbonates moved with the water into the production well zone, where they stimulated the growth of anaerobic prokaryotes (Table 1, Figures S3A,B). The concentrations of acetate and *iso*-butyrate in production water increased from background values 0–5, and 0 in December 2000 to 160.7, and 98.2 mg L^−1^, respectively, in June 2002 (Table 1). Formate, which was undetected in formation water samples before the trial, was registered in June 2002 in water from wells 1002-1, 1008-1, and 1032-1 at concentration 67.4, 35.8, and 1.6 mg L^−1^, respectively. In the zone of production wells, paraffin and asphaltenes content in oil was slightly decreased (Table S1).

These results indicate existence of a geochemically active microbial community in the near-bottom zone of an injection well of a high-temperature oilfield. This community includes cultivable thermophilic and mesophilic aerobic organotrophic bacteria, as well as anaerobic fermenting, sulfate-reducing, and methanogenic microorganisms. Only thermophilic processes were registered in the zone of production wells.

### Statistical analysis of the clone libraries

**Figure 1:**
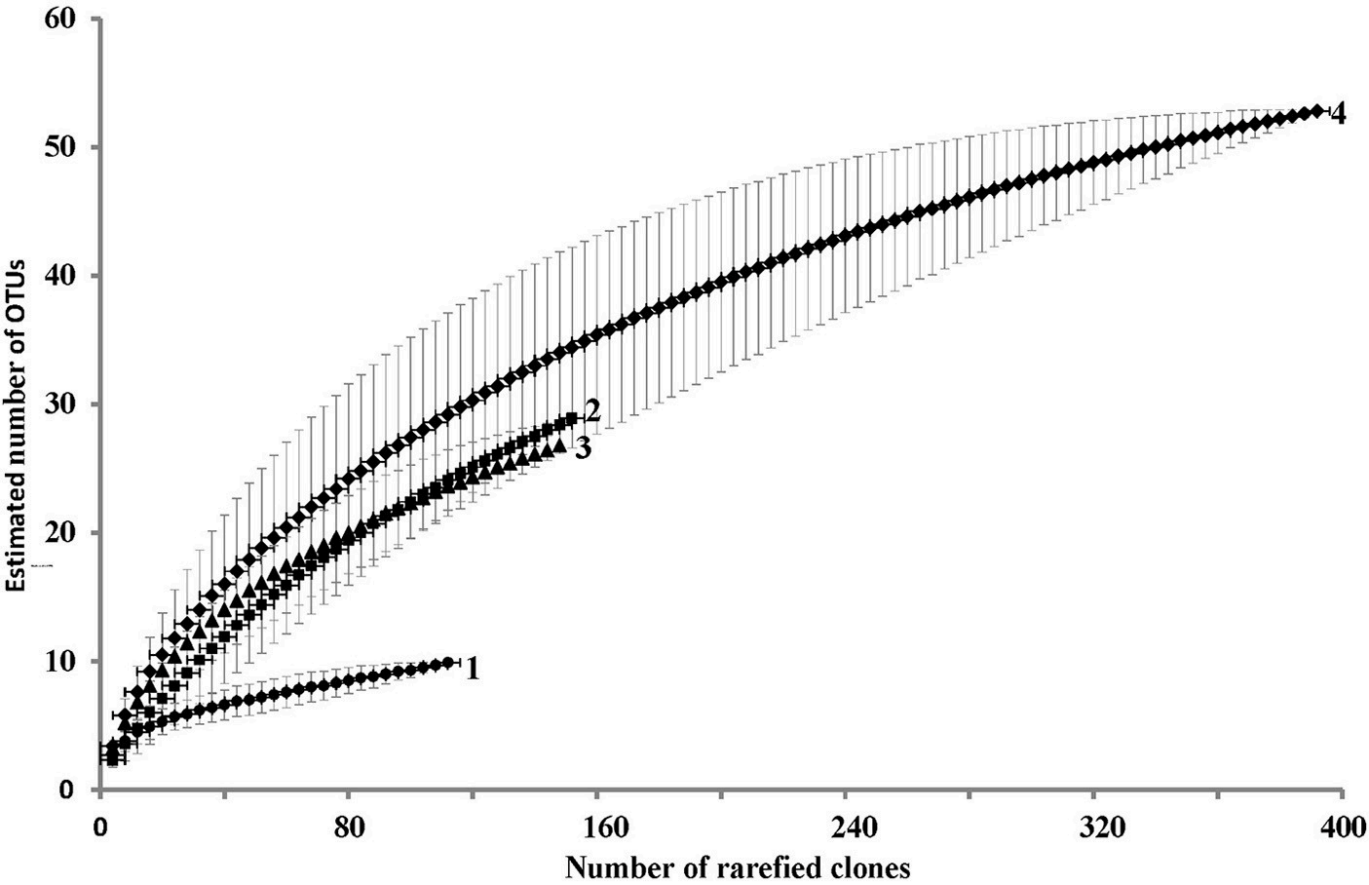
**Rarefaction curves based on the results of comparison of the number of cloned 16S rRNA gene sequences and phylotypes in the DNA-derived clone libraries of *Archaea* (DA library, curve 1, 115 clones), in the DNA-derived library for *Bacteria* (DB, excluding planctomycetes, curve 2, 153 clones), in the RNA-derived library of *Bacteria* cDNA of 16S rRNA sequences (DR, curve 3, 151 clone), and in the total 16S rRNA gene library [DB + DP (89 clones)] + cDNA of 16S rRNA sequences of *Bacteria* (RB), curve 4, 393 clones**. The sequences were grouped in phylotypes at the level of 97% between the sequences of the 16S rRNA genes or cDNA of 16S rRNA transcripts.

Using microbial DNA from water backflushed from the near-bottom zone of injection well 1098 (8 m^3^) and a variety of primers, clone libraries of archaeal (DA) and bacterial, including planctomycetes (DB and DP, respectively) 16S rRNA genes were obtained, as well as the library of the *alkB* genes. The RNA-derived clone library contained only bacterial cDNA of 16S rRNA (RB library) (Figure 1; Table 2; Tables S2, S3). The RNA-derived libraries of archaeal 16S cDNA transcripts and *alkB* genes were not obtained. A total of 551 sequences were analyzed. For statistical analysis, 508 bacterial and archaeal 16S rRNA gene sequences and bacterial cDNA of 16S rRNA sequences were used. The rarefaction curves were close to reaching the plateau (Figure 1). The Good coverage values for the DNA libraries of bacterial 16S rRNA genes (DB) and the RNA library of the cDNA of 16S rRNA clones (RB) were 89.0 and 92.1%, respectively, while the value for the total library of bacterial clones was 95.2%. The coverage value for the archaeal DNA library of the 16S rRNA genes was somewhat higher (95.7%), confirming the representativeness of the clone libraries (Table 2). In all libraries, the species diversity indices were relatively low, although the RNA library was the most diverse one. In the total bacterial library, diversity indices and alignment degree increased, while the domination indices decreased, indicating the difference in predominant phylotypes. Although the Good coverage values for the total library of bacterial clones (DB and RB libraries) and for the archaeal DA library exceeded 95%, the diversity measures presented on Table 2 may be underestimated due to the limited sample size of the clone libraries. However, the advantage of the method of clone libraries analysis used in the present work is that almost complete 16S rRNA gene sequences of *Archaea* and large fragments of bacterial genes were obtained. This is an advantage over current next-generation sequencing approaches, as fragment length and choice of 16S rRNA gene region also affect diversity estimates (Youssef et al., 2009b).

**Table 2:** **Diversity indices of the 16S rRNA gene sequences of *Archaea* and *Bacteria* and cDNA of 16S rRNA sequences of *Bacteria* in the clone libraries generated from DNA and RNA, respectively, of microorganisms of formation water from the high-temperature petroleum reservoir (December 2006)**.

**Parameter**	**Clone library**			
	**DNA-derived**		**RNA-derived**	***Bacteria* total (DB + DP + RB)**
	***Archaea***	***Bacteria***	***Bacteria***	
**Library**	**DA**	**DB**	**RB**	**B_total_**
Number of clones in the library	115	153	151	393
Number of phylotypes and their distribution within the library	10 (44, 36, 16, 7, 7, 1, 1, 1, 1, 1)	29 (100, 9, 4, 3, 3, 3, 3, 3, 2, 2, 2, 2, 1, 1, 1, 1, 1, 1, 1, 1, 1, 1, 1, 1, 1, 1, 1, 1, 1)	27 (44, 36, 10, 10, 6, 6, 5, 5, 4, 3, 2, 2, 2, 2, 2, 1, 1, 1, 1, 1, 1, 1, 1, 1, 1, 1, 1)	52 (100, 57, 45, 37, 26, 11, 10, 10, 6, 6, 6, 5, 5, 4, 3, 3, 3, 3, 3, 3, 3, 3, 2, 2, 2, 2, 2, 2, 2, 2, 2, 2, 2, 1, 1, 1, 1, 1, 1, 1, 1, 1, 1, 1, 1, 1, 1, 1, 1, 1, 1, 1)
Coverage (%)	95.7	89.0	92.1	95.2
Shannon-Weaver diversity index (H)	1.55	1.71	2.40	2.80
Evenness (H/Hmax)	0.67	0.51	0.73	0.71
Simpson's diversity index (1/S)	3.68	2.30	6.31	8.60
Chao1 (min, max 95% confidence interval)	20 (12, 62)	65 (39, 156)	41 (31, 82)	66 (57, 95)
Berger-Parker Dominance index (D)	0.38	0.65	0.29	0.25

### Diversity of 16S rRNA genes of *Archaea* and *Bacteria* and *alkB* genes in the DNA-derived clone libraries generated from back-flushed water

#### Diversity of 16S rRNA genes of *Archaea*

**Figure 2:**
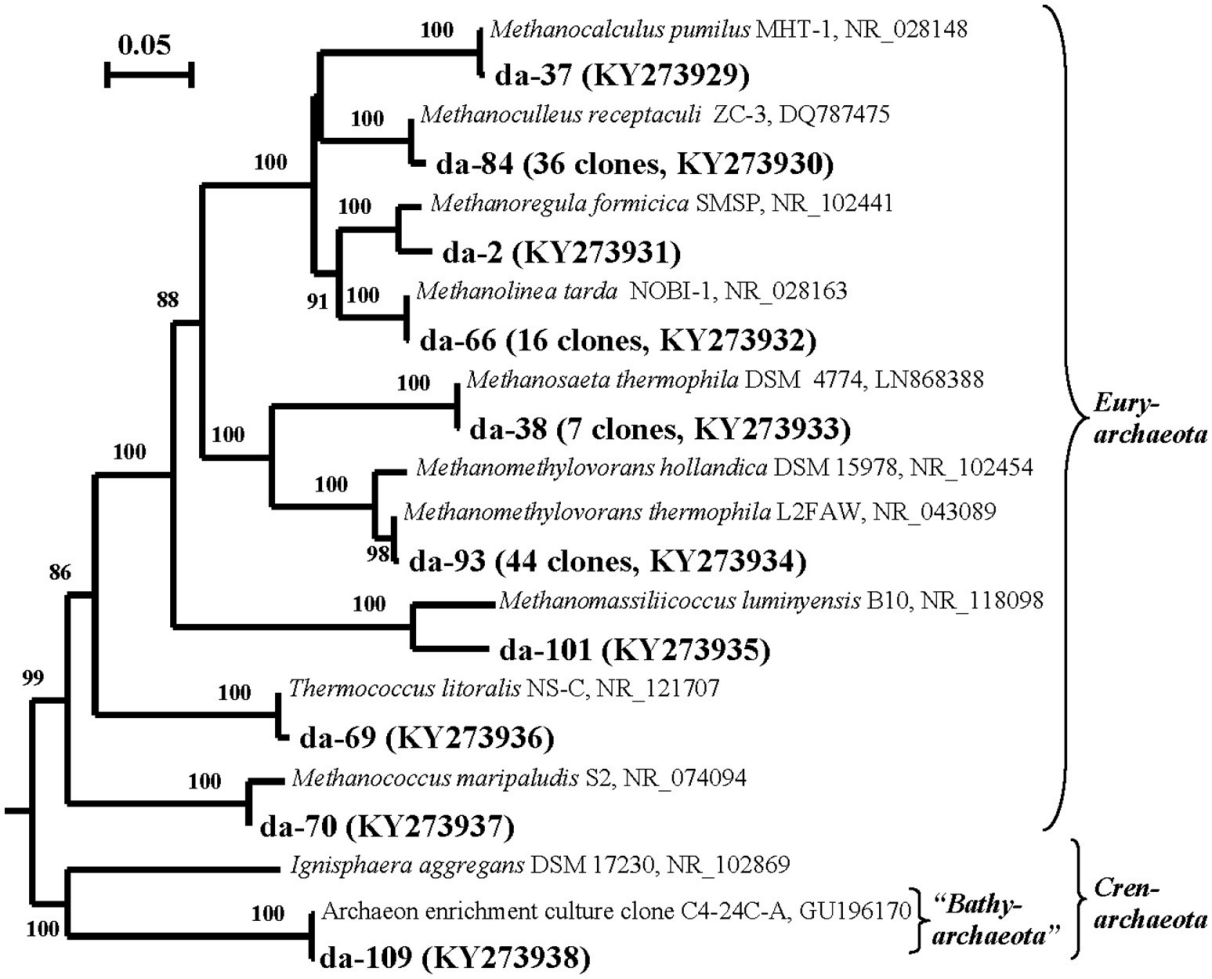
**Phylogenetic neighbor-joining tree constructed on the basis of analysis of the 16S rRNA gene sequences of *Archaea* revealed in the DNA-derived clone library of formation water from near-bottom zone of injection well 1098-8 m^3^ sampled in December 2006 from the Kongdian bed**. Designation: DA means DNA-based library of *Archaea* clones (115 clones). Scale bars show five (0.05) nucleotide substitution per 100 nucleotide base pairs. The numerals at the branching points show the significance of the branching order as determined by bootstrap analysis of 100 alternative trees (only bootstrap values above 85% were considered significant).

Archaeal 16S rRNA genes were detected only in the DNA preparation obtained from back-flushed water, but not in the RNA preparation. The DNA-derived (DA) library contained 115 clones with the inserts of archaeal 16S rRNA genes. The sequences formed 10 phylotypes, of which one belonged to the phylum “*Bathyarchaeota*” (0.9%) and the others, to the phylum *Euryarchaeota* and belonged to six orders: *Methanomicrobiales* (52.2% of the total clone number in DA library), *Methanosarcinales* (44.3%), *Methanomassiliicoccales* (0.9%), *Methanococcales* (0.9%), and *Thermococcales* (0.9%) (Figure 2; Table S2 and Figure S4A).

#### Diversity of 16S rRNA genes of *Bacteria*

**Figure 3:**
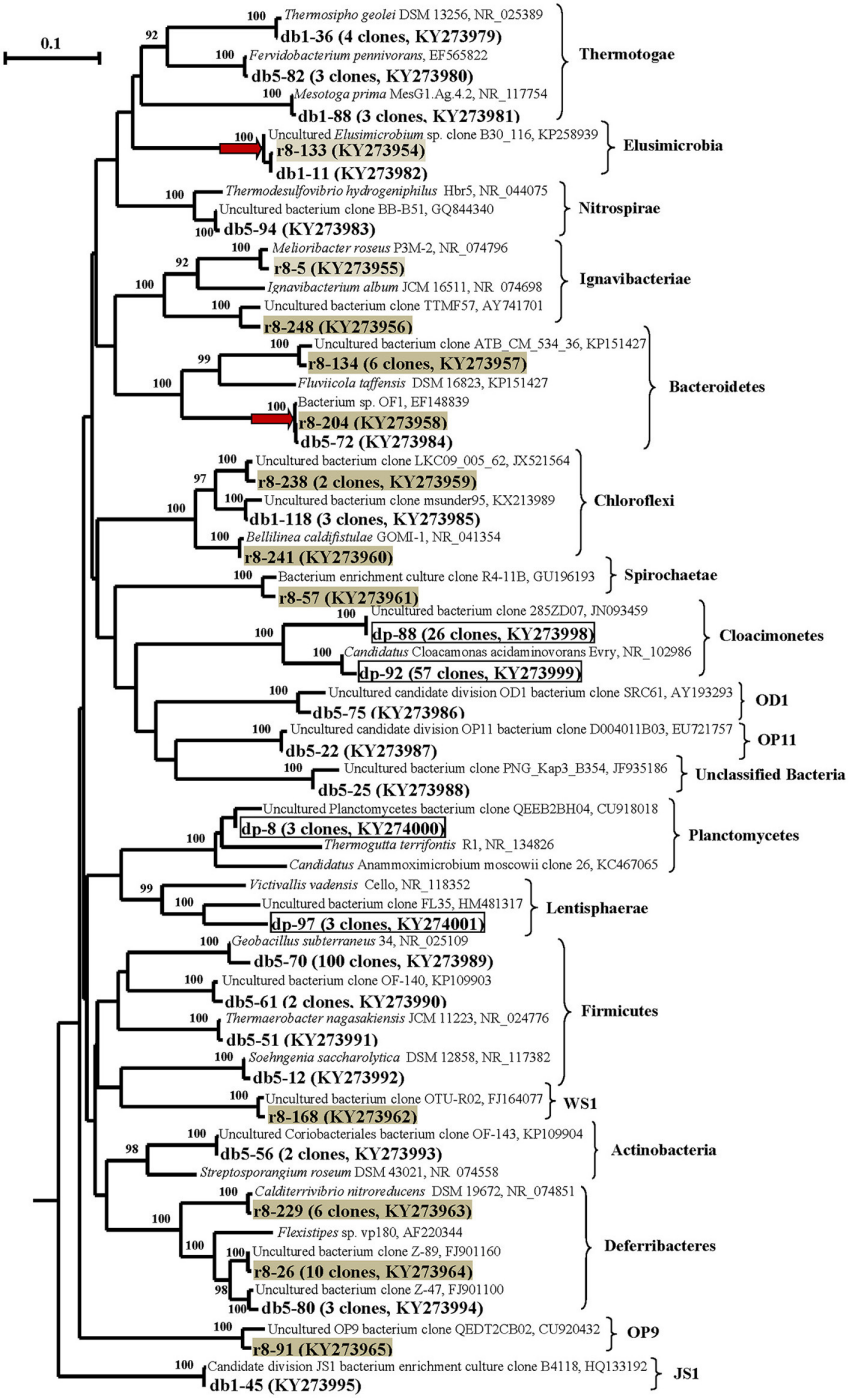
**Phylogenetic neighbor-joining tree constructed on the basis of analysis of the 16S rRNA gene and cDNA of 16S rRNA sequences of *Bacteria* (excluding *Proteobacteria*) revealed in the DNA- and RNA-derived clone libraries of formation water from near-bottom zone of injection well 1098-8 m^3^ sampled in December 2006 from the Kongdian bed**. Designation: DB means DNA-based library, *Bacteria* clones (153 clones), DP—DNA-based library of *Bacteria* clones obtained with primers specific for *Planctomycetes* (89 clones) (marked by frame), RB—RNA-based library of *Bacteria* cDNA of 16S rRNA sequences (151 clones) (marked by gray filling). The clusters common for DB and RB-libraries are marked by red arrow. Scale bars show 10 (0.1) nucleotide substitution per 100 nucleotide base pairs. The numerals at the branching points show the significance of the branching order as determined by bootstrap analysis of 100 alternative trees (only bootstrap values above 85% were considered significant).

**Figure 4:**
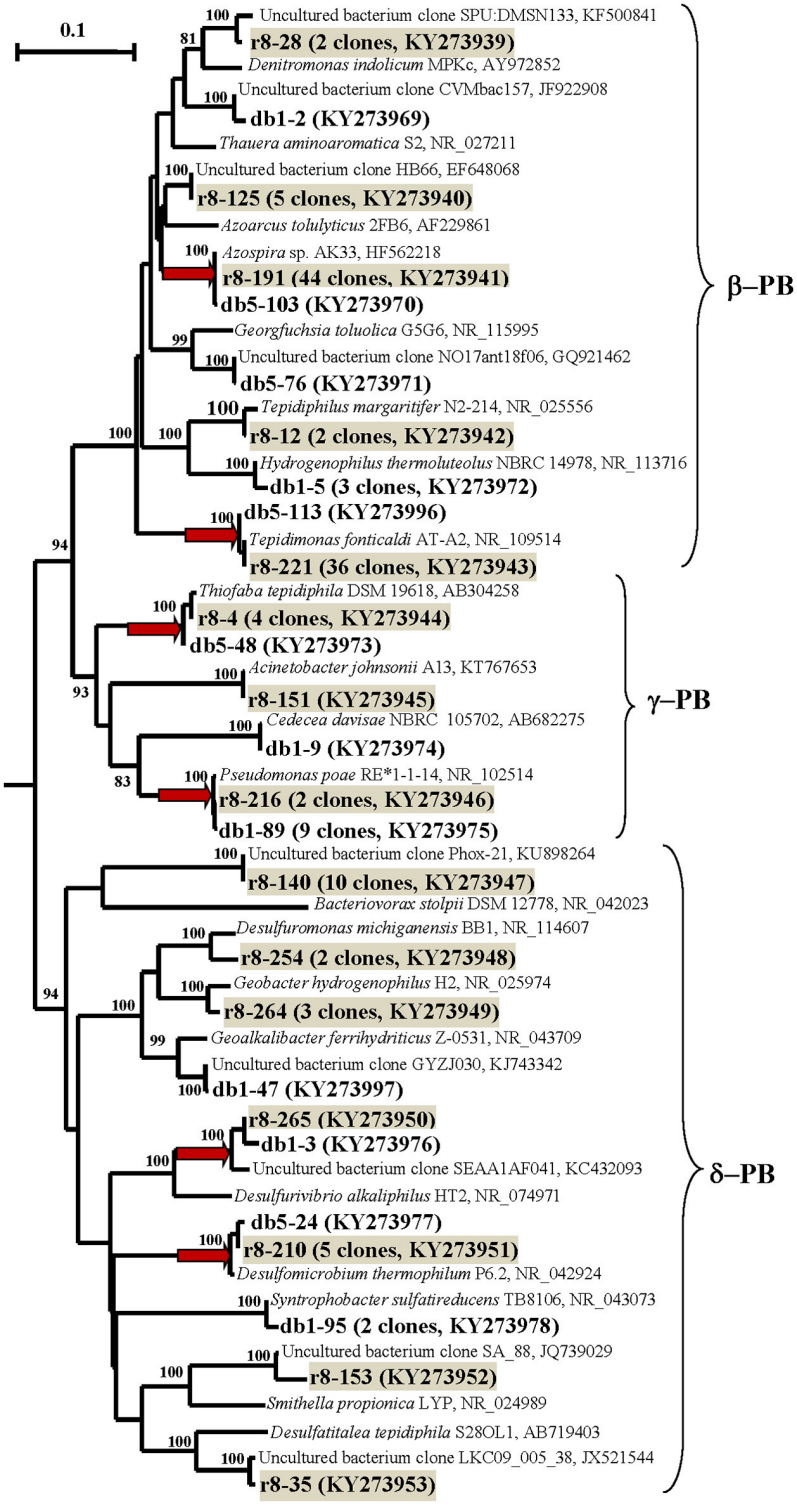
**Phylogenetic neighbor-joining tree constructed on the basis of analysis of the 16S rRNA gene and cDNA of 16S rRNA sequences of *Proteobacteria* revealed in the DNA- and RNA-derived clone libraries of formation water from near-bottom zone of injection well 1098-8 m^3^ sampled in December 2006 from the Kongdian bed**. The designations are as on Figure 3.

The DNA-derived DB and DP libraries of bacterial 16S rRNA gene clones (including those of planctomycetes) obtained using two primer pairs (8–27f–519r and Pla46f–519r) contained 153 and 89 clones, respectively (Figures 3, 4; Table S2 and Figures S4B,C).

In the case of the DB library, 153 sequences were grouped into 29 phylotypes, belonging mostly to members of the phyla *Firmicutes* (67.9% of the total clone number in DB library) and *Proteobacteria* (15.0%). The sequences belonged to the classes *Bacilli* (65.3%), *Thermotogae* (6.5%), γ-*Proteobacteria* (7.1%), β-*Proteobacteria* (4.5%), δ-*Proteobacteria* (3.3%), *Deferribacteres* (2.0%), and *Chloroflexi* (2.0%). The minor phylotypes belonged to *Actinobacteria, Nitrospira, Bacteroidetes, Elusimicrobia, Clostridia, Tissierellia*, and to uncultured bacteria of the OD1, OP11, and JS1 phylogenetic lineages (9.1%) (Figures 3, 4; Table S2 and Figure S4B).

Most of the *Firmicutes* genes belonged to aerobic thermophilic spore-forming bacteria of the genus *Geobacillus* (65.3% of the clones in the DB library).

The second most numerous group consisted of the sequences of the γ-*Proteobacteria* related to the 16S rRNA sequence of a mesophilic aerobic organotrophic bacterium *Pseudomonas poae*. The β-*Proteobacteria* sequences retrieved belonged to aerobic thermophilic organotrophic bacteria of the genus *Tepidimonas* and to the hydrogen- and/or sulfur-oxidizing bacteria of the genus *Hydrogenophilus*; some of minor sequences were similar to those of the mesophilic organotrophic bacterial genera *Thauera, Azospira*, and *Georgfuchsia* (Figure 4; Table S2 and Figure S4B). The δ-*Proteobacteria* phylotypes were located in the clusters of the genes of the thermophilic sulfate-reducing bacteria *Desulfomicrobium thermophilum*, mesophilic sulfate- and sulfur-reducing bacteria *Syntrophobacter* and *Desulfurivibrio*, and iron-reducing bacteria of the genus *Geoalkalibacter*.

The *Thermotogae* sequences belonged to thermophilic fermentative bacteria *Thermosipho geolei* and *Fervidobacterium pennivorans* and to mesophilic *Mesotoga prima*. The *Nitrospira* sequences exhibited 95% similarity to the gene of the thermophilic sulfate-reducing bacterium *Thermodesulfovibrio hydrogeniphilus*.

Special attention was focused on *Planctomycetes*, since the 16S rRNA genes of this group have been revealed in the library of Kongdian oilfield microbial community (Nazina et al., 2006), as well as in 27% of clone libraries from hydrocarbon-impacted environments (Gray et al., 2010). *Planctomycetes* diversity and activity in oilfields remains insufficiently studied. In spite of the application of planctomycetes-specific primers Pla46f–519r, only one of the four phylotypes in the DP library (3 out of 89 clones) belonged to members of the phylum *Planctomycetes* and was distantly related to the genes of thermophilic facultatively anaerobic bacteria of the genus *Thermogutta* (90% similarity) (Figure 3, Table S2 and Figure S4C). Over 93.2% of the sequences belonged to uncultured bacteria of the candidate phylum *Cloacimonetes* (WWE1) (Dojka et al., 1998; Rinke et al., 2013), including Candidatus “*Cloacamonas acidaminovorans*” (99% сходства). The remaining sequences of the DP library were remotely related to the 16S rRNA genes of bacteria of the class *Lentisphaerae*. Although the Pla46 primer was initially developed as the one specific for the phylum *Planctomycetes* (Neef et al., 1998), our analysis of coverage using the TestPrime tool on the SILVA website revealed the specificity of amplification of the 16S rRNA gene fragments by the Pla46–1390r primer pair for planctomycetes to be ~44.0%. These primers were, however, specific for some new bacterial phyla, including *Lentisphaerae* (74.1%) and the Candidate division *Cloacimonetes* (60.6%), which agrees with the results of our analysis of the clone library.

#### Diversity of alkB genes

The library of the *alkB* sequences contained 43 genes, which formed three phylotypes. Two phylotypes were closely related to the homologs of the *alkB-geo1* (22 out of 43 clones) and *alkB-geo6* genes (5 out of 43 clones) (Table S2). The third phylotype (16 clones) exhibited only 84% similarity to the *alkB* genes of cultured bacteria and 98% similarity to the *alkB* genes revealed in the microbial community of formation water from production wells at the onshore Potiguar Basin (Northeast, Brazil) (Verde et al., 2013) and of oil exploring area located in the Xinjiang Uygur Autonomous Region (Northwest, China) (Yang et al., 2015). The third phylotype probably belongs to a presently unknown microorganism inhabiting oilfields.

### Diversity of cDNA of 16S rRNA sequences of *Bacteria* in the RNA-derived clone library generated from back-flushed water

The RNA-derived RB library contained 151 cDNA of 16S rRNA sequence of bacteria belonging to 27 phylotypes (Figures 3, 4; Table S3 and Figure S4D). The sequences of metabolically active β-, δ-, and γ-*Proteobacteria* were responsible for 58.9, 15.2, and 4.6% of the total sequence number in the RB library, respectively. The remaining sequences belonged to members of the *Deferribacteres* (10.6%), *Bacteroidetes* (4.6%), *Chloroflexi* (1.9%), *Ignavibacteriae* (1.3%), *Elusimicrobia* (0.6%), and uncultured bacterial lineages (1.9%).

The most numerous sequences in the RB library belonged to the β-*Proteobacteria:* aerobic thermophilic *Tepidimonas fonticaldi* (23.8% clones in RB; 100% similarity) and a mesophilic bacterium distantly related to *Thauera aminoaromatica* (29.1% clones; 92% similarity). Some phylotypes exhibited low similarity to the genes of *Azoarcus tolulyticus* (95%) and *Denitromonas indolicum* (94%). The minor phylotypes belonged to the thermophilic bacterium *Tepidiphilus margaritifer* (99%), which is capable of organotrophic growth either aerobically of anaerobically with nitrate.

The retrieved sequences of the δ-*Proteobacteria* included the genes of a thermophilic sulfate-reducing bacterium *Desulfomicrobium thermophilum* (99% similarity) and of mesophilic sulfate reducers (genus *Desulfatitalea*, 90%), of sulfur-reducing (genera *Desulfuromonas*, 96% and *Desulfurivibrio*, 92%) and iron-reducing bacteria (*Geobacter hydrogenophilus*, 97%), as well as of an uncultured bacterium remotely related to a syntrophic bacterium *Smithella* sp. The sequences of the γ-*Proteobacteria* were related (99%) to the genes of the known aerobic mesophilic organotrophic bacteria *Pseudomonas* and *Acinetobacter*, which are common in oilfields, and of a thermotolerant autotrophic sulfur-oxidizing bacterium *Thiofaba tepidiphila* (Mori and Suzuki, 2008).

The *Deferribacteres* sequences, which were numerous in the RB library, belonged to an obligately anaerobic, moderately thermophilic bacterium *Calditerrivibrio nitroreducens* (99%), which reduces nitrate to ammonium when grown on acetate, pyruvate, lactate, or yeast extract (Iino et al., 2008), as well as to bacteria distantly related to *Flexistipes* sp.

The sequences of metabolically active *Chloroflexi* belonged to *Bellilinea caldifistulae* (99%) (Yamada et al., 2007), anaerobic filamentous thermophilic fermenting bacteria of the class *Anaerolineae*, which are common inhabitants of hydrocarbon-contaminated ecosystems, oilfields, and methanogenic cultures grown on *n*-alkanes and volatile acids (Dojka et al., 1998; Liang et al., 2015).

The sequences of a unique thermotolerant bacterium *Melioribacter roseus*, belonging to a new phylum *Ignavibacteriae* (Podosokorskaya et al., 2013), were also revealed.

## Discussion

### Biogeochemical processes and cultivable microorganisms in formation water from the near-bottom zone of an injection well and from production wells of the kongdian bed

Water flooding, which is used to maintain the reservoir pressure and enhance oil recovery, results in delivery of oxidizers and microorganisms in the oilfield and changes the conditions in the near-bottom zone of injection wells. In the present work, microbial diversity and activity was studied in the near-bottom zone of an injection well at the Kongdian bed of the Dagang oilfield, which has been water-flooded with produced water from the same oilfield for over 30 years. Back-flushed water was sampled during the application of a biotechnology for enhanced oil recovery based on injection of nitrogen and phosphorus salts together with a water-air mixture and/or a hydrogen peroxide solution as an oxygen source.

Prokaryotic functional diversity in environments has recently often been studied using molecular techniques alone. The functional role of a specific microbial group in a reservoir is then determined by extrapolation of the known data on the metabolic potential of the group revealed by analysis of the 16S rRNA genes or of the functional genes, which we consider an unsatisfactory conclusion. Orphan et al. (2003) compared the geochemical conditions and results of molecular investigation of two high-temperature oilfields and also concluded “that the community structure of the free-living assemblage characterized with molecular methods and the metabolic potential of the resident community may differ dramatically” (Orphan et al., 2003). The necessity of comprehensive investigation of the physicochemical and geological conditions in the environment and microbial abundance and activity using all available up-to-date methods was stated by the founders of geological microbiology, S.I. Kuznetsov, M.V. Ivanov and N.N. Lyalikova in their well-known monograph “Introduction in geological microbiology” (1963).

In the present work the composition of the microbial community from water of the near-bottom zone of injection well at the Kongdian bed was studied by comparative analysis of the DNA and RNA libraries of the 16S rRNA gene clones and cDNA of 16S rRNA transcripts and of the *alkB* functional gene and the results of investigation were then confirmed by the data obtained by cultural, physicochemical, radioisotope, and stable isotope research.

Delivery of the oxidizers and cooled water into the bed resulted in development of an oil-oxidizing aerobic/anaerobic community in the near-bottom zone of the injection well. The cultivable components of this community were thermophilic and mesophilic aerobic organotrophic bacteria, including hydrocarbon oxidizers, as well as fermenting, sulfate-reducing, and methanogenic prokaryotes (Figure S2A). The processes of thermophilic and mesophilic sulfate reduction and methanogenesis were registered by radiotracer analysis in the near-bottom zone, while thermophilic processes occurred in the area of production wells. In the absence of sulfate, methanogenesis was the major anaerobic terminal process of oil biodegradation in the area of production wells (Figures S2B,C). Increase of sulfates in the course of the biotechnological treatment resulted in a considerable intensification of sulfate reduction in the near-bottom zone. Both sulfate reduction and methanogenesis were detected in the same water samples and probably occurred simultaneously in different microniches within the oil bed. We have previously shown that methane was formed in the Kongdian bed due to both CO_2_ reduction with hydrogen and syntrophic decomposition of acetate by a consortium of *Thermoanaerobacter ethanolicus* and *Methanothermobacter thermautotrophicus* (Shestakova et al., 2011a).

The averaged composition of oil from the Dagang oilfield provided by the oil-producing company was cited in our previous publications (Nazina et al., 2006, 2008). In the course of the present work, oil density in the Kongdian bed was found to vary from 0.9459 to 0.9549 g/cm^3^, indicating it being heavy oil (Table S1). Oil biodegradation in the zone of the production well 1002-1 was accompanied by a slight decrease in its viscosity, as well as in the content of paraffin and asphaltenes.

Injection of H_2_O_2_ solution instead of the water-air mixture resulted in suppression of both aerobic and anaerobic processes in the near-bottom zone, although microbial viability was not affected, which was indicated by high numbers of cultivable microorganisms of various physiological groups (Table 1, well 1008-1, Dec., 2006). Increased concentrations of bicarbonate, CO_2_, acetate, *iso*-butyrate, and formate in formation water indicated biological transformation of oil organic matter in the oilfield. Oil biodegradation in formation water of the near-bottom zone resulted in elevated levels of isotopically light mineral carbonates, with δ^13^C values [δ^13^C/∑(CO_2_ + ${\text{HCO}}_{3}^{-}$ + ${\text{CO}}_{3}^{2-}$)] decreasing from 4.2‰ to −0.4…−8.5‰ PDB at some stages of the treatment (Table 1).

Biodegradation of the aromatic and polyaromatic fractions of oil with formation of methane was also shown for anaerobic microcosms from the Dagang oilfield incubated at 55–60°C (Jiménez et al., 2012). Consumption of oil *n*-alkanes and polyaromatic components by aerobic mesophilic enrichments obtained from injected and formation water of the Dagang oilfield in the medium with oil was also shown (Cai et al., 2015).

Injection of dissolved oxygen or H_2_O_2_ was accompanied by microbial oxidation of iron sulfides, which are usually present in sandy oil-bearing strata, with formation of sulfate (12−135 mg l^−1^) and ferric iron. The latter was responsible for the characteristic orange precipitate in backflushed water.

The mineral salts NH_3_-N and ${\text{PO}}_{4}^{3-}$ injected into the oil field were detected in small concentrations in the water of practically all production wells (NH_3_-N was in a range 1−17 mg l^−1^, ${\text{PO}}_{4}^{3-}$ − 0−0.8 mg l^−1^). Although neither injected water nor formation water contained oxidized nitrogen compounds, during the trial nitrate and nitrite were detected in the near-bottom zone of the injection well (0.07 and 0.18 mg l^−1^, respectively) and in the water from 20 production wells (0.02−0.34 and 0.07−0.55 mg l^−1^, respectively, indicating activity of the microorganisms of the nitrogen cycle (Nazina et al., 2008).

Interestingly, while the sample from well 1008-1 (Dec., 2006) contained cultivable sulfate-reducing bacteria (≥10^5^ cells/mL), sulfate reduction was not detected. Thus, even detection of cultivable bacteria is not always an unequivocal indication of their metabolic activity.

Development of an aerobic-anaerobic community including the microorganisms of the carbon, sulfur, iron, and nitrogen cycles in the near-bottom zone of the injection well was confirmed by investigation of the DNA- and RNA-derived clone libraries of the 16S rRNA genes and 16S cDNA transcripts, respectively.

### Comparative analysis of phylotypes in DNA- and RNA-derived clone libraries

Next-generation sequencing molecular approach certainly results in more complete assessment of microbial diversity than analysis of the clone libraries. In the few works where the data on bacterial 16S rRNA genes obtained from oilfields using DNA pyrosequencing and gene clone library approaches was compared, the results of statistical analysis (rarefaction curves) and the predominant bacteria detected by these two approaches were identical (Wang et al., 2014). DNA isolation from oil-contaminated samples may be difficult, since residual oil prevents the isolation of nucleic acids. Accurate comparison of the DNA- and RNA-based libraries required using a single method (either cloning or high-throughput sequencing). The amount of RNA isolated in the course of our experiments was very low. Since RNA-based investigation of the composition of the microbial community in oil-containing samples has not been attempted previously, the tested cloning approach was chosen for the purpose.

Archaeal RNA-derived clone library of cDNA of 16S rRNA was not obtained in the present work. In the DNA-derived DA clone library, thermophilic methylotrophic methanogens *Methanomethylovorans thermophila*, which grow within a broad temperature range (42–58°C), were responsible for 38.2% of the 16S rRNA gene sequences. The genes of thermophilic (*Methanoculleus receptaculi*, 31.3% and *Methanolinea tarda*, 13.9%) and mesophilic (*Methanocalculus pumilus*, 6.1% and *Methanococcus maripaludis*, 0.9%) hydrogen- and formate-utilizing methanogens were also well represented. The sequences of acetate-utilizing methanogens belonged to the thermophilic *Methanothrix soehngenii* (previously 2*Methanosaeta thermophila*, 6.1%). A minor phylotype belonged to *Thermococcus litoralis*, a thermophilic archaeon with fermentative metabolism, which has been previously isolated from high-temperature oil fields of the North Sea and Alaska (Stetter et al., 1993). Most of revealed archaea were adapted to the conditions of water-flooded oil fields and have been repeatedly isolated from these environments.

The DNA- and RNA-derived clone libraries of the *Bacteria* domain were complementary, with eight phylotypes revealed in both libraries (Figures S4B–E). Both libraries were found to contain the sequences of aerobic organotrophic bacteria (genera *Pseudomonas, Tepidimonas*), denitrifying (*Azospira*), sulfate-reducing (*Desulfomicrobium*), sulfur-reducing (*Desulfurivibrio*), and sulfur-oxidizing bacteria (*Thiofaba*), as well as uncultured *Elusimicrobium* and *Bacteroidetes* with unknown metabolic functions. The sequences of some metabolically active bacteria belonged to organotrophs of the genus *Acinetobacter*, which has been repeatedly isolated from oilfields, and to iron-reducing bacteria *Geobacter*.

Analysis of the DNA- and RNA-derived libraries resulted in more complete detection of the members of the major functional groups. Our data indicate the functioning of both thermophilic and mesophilic variants of the trophic chain in the near-bottom zone of the injection well.

*Aerobic hydrocarbon-oxidizing bacteria* are responsible for the initial stage of oil biodegradation in the presence of dissolved oxygen (air or H_2_O_2_). The functional analogs of thermophilic aerobic organotrophic, hydrocarbon-oxidizing bacteria (genera *Geobacillus, Tepidimonas, Tepidiphilus*, and *Thermaerobacter*) are mesophilic *Pseudomonas, Acinetobacter*, and *Shewanella;* the latter was previously isolated from this oilfield (Shestakova et al., 2011b). These organisms are known to oxidize both the saturated *n*-alkanes and aromatic hydrocarbons of oil.

The physiological characteristics of *Tepidimonas fonticaldi*, which was revealed in both libraries, were in the best agreement with the conditions of the near-bottom zone. This bacterium grows aerobically on organic acids (acetate, lactate, and pyruvate) and amino acids within a broad temperature range from 35 to 60°C (with the optimum at 55°C), at low ambient salinity and neutral pH, and is capable of nitrate reduction (Chen et al., 2013). No data are presently available concerning its ability to utilize components of crude oil.

Pure cultures of hydrocarbon-oxidizing geobacilli, including the new species *Geobacillus jurassicus*, have been repeatedly isolated from the Kongdian oilfield (Nazina et al., 2005; Shestakova et al., 2011b). Earlier analysis of the DNA- and RNA-derived clone libraries obtained from an enrichment culture of aerobic hydrocarbon-oxidizing bacteria of the near-bottom zone of injection well 1098, Kongdian oilfield, revealed predominance of *Geobacillus subterraneus* and *Aeribacillus* (previously *Geobacillus*) *pallidus* in this enrichment (Shestakova et al., 2011b).

The genes *alkB-geo1* and *alkB-geo6*, related to homologs of the *alkB* gene of geobacilli, were present in the *alkB* gene library. The *alkB-geo1* homolog is universal for a number of strains of hydrocarbon-oxidizing geobacilli (Tourova et al., 2008). The *alkB-geo1* and *alkB-geo6* homologs are related to the *alkB4* and *alkB2* genes, respectively, of *Rhodococcus erythropolis* NRRL B-16531. It should be noted that the 16S rRNA gene sequences of rhodococci, unlike those of geobacilli, were not revealed in our libraries. Thus, it may be concluded that the *alkB-geo1* and *alkB-geo6* sequences in this library belong to geobacilli.

In spite of numeric predominance of the 16S rRNA genes of the genus *Geobacillus* in the DNA library (DB) and detection of the sequences of the *alkB-geo1* and *alkB-geo6* functional genes (probably belonging to geobacilli), no cDNA of 16S rRNA sequences of this genus were revealed in the RNA-derived library (RB). The water sample from the near-bottom zone of the injection well (1098-8 m^3^) was collected after injection of H_2_O_2_ solution. Oxic conditions could probably suppress growth of the numerous geobacilli and result in decreased rates of sulfate reduction and methanogenesis in this zone, as well as in the absence of the cDNA of 16S rRNA sequences of metabolically active archaea in the RB library.

Members of the genera *Pseudomonas* and *Thauera* are able to grow on aromatic hydrocarbons either aerobically or via nitrate reduction. Although nitrate concentration in the Kongdian formation water was low, denitrifying activity of *Pseudomonas* and *Thauera* in the near-bottom zone of the injection well, as well as aerobic growth by oxidation of oil aromatic hydrocarbons, cannot be ruled out. Recent investigation of microorganisms from three oilfields by analysis of the *napA* functional gene encoding a nitrate reductase subunit revealed predominance in the *napA* libraries of the genes of nitrate-reducing bacteria of the orders *Burkholderiales, Rhodocyclales* (genera *Azoarcus* and *Thauera*), and *Pseudomonadales* (genus *Pseudomonas*) (Feng et al., 2011).

*Fermenting prokaryotes*, similar to aerobic organotrophs, were represented by thermophilic bacteria (genera *Thermosipho, Fervidobacterium, Bellilinea*) and archaea (genus *Thermococcus*) and mesophilic/thermotolerant bacteria (genera *Soehngenia, Mezotoga, Flexistipes*). Depending on the ecological conditions, thermophilic fermenting bacteria, such as *Thermoanaerobacter*, which has been previously isolated from the Kongdian oilfield, may function as syntrophic bacteria when grown with methanogens on acetate (Shestakova et al., 2011a) and to be functional analogs of mesophilic syntrophic bacteria (genera *Smithella, Syntrophobacter*, and *Soehngenia*). Members of the genus *Smithella* were revealed in anaerobic microbial communities degrading *n*-alkanes, oil, and lower fatty acid in syntrophy with hydrogen-utilizing methanogens (Gieg et al., 2008; Jones et al., 2008; Gray et al., 2010).

*Thermosipho geolei* (*Thermotogae*) has been previously isolated from the Samotlor high-temperature oilfield (L'Haridon et al., 2001). What substrates are used by these fermenting bacteria in the oilfield, where carbohydrates, their preferable substrate, are absent, remains unclear. In the trophic chain they may act as degraders of microbial biomass, or degrade organic compounds of oil as components of a community, and/or grow as sulfidogens with molecular hydrogen, reducing elemental sulfur to sulfide.

It should be noted that thermophilic fermenting bacteria of the genus *Thermotoga* and archaea of the genus *Thermococcus* are able to reduce Fe^3+^ when growing on hydrogen (Slobodkin et al., 1999).

*Iron-reducing bacteria* (genus *Shewanella*) have been detected in formation water of oilfields and in microbial communities degrading oil, long-chain C_24_–C_34_ *n*-alkanes, and aromatic compounds (Kato et al., 2001). We have isolated pure cultures of *Shewanella putrefaciens* from near-bottom water of a Kongdian injection well. This organism degraded oil *n*-alkanes under oxic conditions and grew on acetate reducing Fe^3+^ to Fe^2+^ under anoxic conditions (Shestakova et al., 2011b). Investigation of pure cultures, together with the results of molecular research and detection of a phylotype of an iron-reducing bacterium *Geobacter hydrogenophilus* (r8-264, Table S2), which is capable of growth on acetate and aromatic substrates, indicate the possible functioning of the geochemical cycle of iron coupled to oil biodegradation in the injection well area.

The cDNA of 16S rRNA sequences of aerobic and anaerobic *bacteria of the sulfur cycle* were abundant in the RB library. Detection of the cDNA of 16S rRNA sequences of sulfur-oxidizing bacteria (*Thiofaba*) in the library of metabolically active prokaryotes was supported by the presence of sulfate (up to 135 mg l^−1^) during injection of the oxidizers with a consequent increase in abundance of cultured sulfate-reducing bacteria, detection of the sequences of sulfate-, sulfite, and sulfur-reducing bacteria (genera *Desulfomicrobium, Thermodesulfovibrio*, and *Desulfurivibrio*) in the libraries, and of mesophilic and thermophilic sulfate reduction in the studied formation water sample (1098-8 m^3^) (Table 1, Dec., 2006, Figure S2B). The presence of sulfate-reducing bacteria in the absence of sulfate in formation water may be explained by their ability to function as proton-reducing acetogens or fermenters. The sequences of *Thermotogae*, syntrophic, and sulfate-reducing bacteria have been repeatedly revealed by molecular investigation of formation water from different oilfields (Gray et al., 2010).

The bacterium Candidatus “*Cloacamonas acidaminovorans*,” which was revealed by analysis of the DNA-derived DP library, possesses the key enzymes of amino acid metabolism and is a *syntrophic bacterium* (Pelletier et al., 2008). In the near-bottom zone this bacteria may be involved in both syntrophic degradation of organic matter and degradation of the components of microbial biomass.

The phylotypes of uncultured bacterial lineages OD1, OP9, OP11, and JS1 were detected previously in libraries of oilfield microorganisms (Gittel et al., 2009) and in hexadecane-utilizing methanogenic consortia. Members of the phylum OP11 were revealed in 6 out of 26 analyzed 16S rRNA gene clone libraries of microorganisms from the surface and subterranean hydrocarbon-containing habitats (Gray et al., 2010). Members of the lineages OP9 and JS1 (candidate phylum “Atribacteria”) are widespread in anoxic marine sediments, geothermal ecosystems, and oilfields and probably ferment sugars and/or organic acids (e.g., propionate) (Nobu et al., 2016).

Thus, comparative analysis of the DNA- and RNA-derived libraries revealed the components of microbial community, which would have remained undetected by analysis of either the DNA- or the RNA-derived clone library alone.

In our opinion, the taxonomic position of microorganisms revealed in a habitat is less important from the ecological point of view than their position as members of a functional group within the trophic chain. Our results on the functional and phylogenetic microbial diversity in different zones of a high-temperature oilfield showed that even variations in the temperature mode in the near-bottom zone of the injection well did not result in significant changes in the functional components of the microbial trophic chain. Two branches of the trophic chain formed in the zone of injection of the surface water were represented by thermophilic and mesophilic components of the community, which included aerobic hydrocarbon oxidizers and, sulfide- and Fe^2+^ -oxidizers, as well as anaerobic fermenting, syntrophic, sulfate- and iron-reducing, and methanogenic prokaryotes.

The hypothesis that trophic interactions between pelagic microbes may be organized in a fractal-like manner was recently proposed by Våge and Thingstad (2015). According to their fractal hypothesis of the pelagic microbial food web, different trophic levels are controlled in similar manners within their respective characteristic temporal and spatial scales. An understanding of organization at one level would thus provide a basic understanding of organization at all other levels. If this concept is valid and can be formalized, it would be an innovative way of considering phylogenetic diversity and food web complexity. In our opinion, the results of investigation of the trophic relationships within the microbiota of the near-bottom zone of injection well in a water-flooded high-temperature petroleum reservoir (mesophilic and thermophilic food webs) may be an experimental confirmation of their hypothesis. This hypothesis could be used to model the food webs and to predict the biogeochemical processes in petroleum reservoirs using fractal methods.

Our results confirm the opinion of Kuznetsov et al. (1963) and Orphan et al. (2003) that complementary eco-physiological, microbiological, molecular and biogeochemical studies are promising approaches for linking microbial diversity and microbial functioning in petroleum reservoirs.

## Author contributions

TN, NS, NK, and QF contributed to field work. LM, QF, and NK contributed to physical-chemical analysis. NS, and NK estimated rates of microbial processes, NS, ES, AK, and AP obtained DNA and RNA, performed cloning and sequencing. NS, ES, and TT performed phylogenetic and statistical analyses. TN, NS, TT, and AP designed the work. TN, NS, TT, ES, NK, AK, QF, LM, and AP analyzed the data and wrote the manuscript.

## Funding

Ecological studies on the Dagang oilfield were supported by China National Petroleum Corporation (CNPC, grant DFT04-122-IM-18-20RU), molecular study was supported by the Russian Academy of Sciences (Program “Molecular and Cellular Biology”), summarizing of the results and writing of the article were supported by Russian Science Foundation (grant 16-14-00028).

### Conflict of interest statement

The authors declare that the research was conducted in the absence of any commercial or financial relationships that could be construed as a potential conflict of interest.
