# Supplementary material for: Diversity of Metabolically Active Bacteria in Water-Flooded High-Temperature Heavy Oil Reservoir
Source: Front Microbiol. 2017 Apr 25;8:707. doi: 10.3389/fmicb.2017.00707 (PMC5403907; doi:10.3389/fmicb.2017.00707)
Supplement: Supplementary file 1 [file DataSheet1.pdf]

Supplementary Material

Diversity of metabolically active *Bacteria* in water-flooded high-temperature heavy oil reservoir

Tamara N. Nazina*, Natalya M. Shestakova, Ekaterina M. Semenova, Alena V. Korshunova, Nadezda K. Kostrukova, Tatiana P. Tourova, Liu Min, Qingxian Feng, and Andrey B. Poltaraus

[1] *Laboratory of Petroleum Microbiology, Winogradsky Institute of Microbiology, Research Center of Biotechnology, Russian Academy of Sciences, Moscow, Russia*
[2] *Dagang Oil Field Group Ltd., Tianjin, China*
[3] *Engelhardt Institute of Molecular Biology, Russian Academy of Sciences, Moscow, Russia*

* Correspondence: Tamara N. Nazina, Laboratory of Petroleum Microbiology, Winogradsky Institute of Microbiology, Research Center of Biotechnology, Russian Academy of Sciences, Prospect 60-Letiya Oktyabryua, 7/2, 117312 Moscow, Russia. Tel.: +7-499-135-03-41
e-mail: nazina@inmi.ru

Supplementary tables

Supplementary Table S1. Density, viscosity, wax and colloidal asphaltene content of oil from production well 1002-1 at the Kongdian bed of the Dagang oilfield in the course of the pilot trial.

Parameter	Year, month					
	2001.03	2001.08	2002.04	2002.11	2003.04	2003.09
Viscosity, mPa.s	501.2	496.5	458.9	459.1	470.3	465
Density, g/cm^3	0.9549	0.9547	0.9459	0.9462	0.9528	
Wax content, %	6.64	6.54	6.48			4.72
Colloidal asphaltene, %	41.05	39.81	37.21			38.88

Supplementary Table S2. Phylogenetic diversity of 16S rRNA genes of *Archaea* and *Bacteria* and *alkB* genes detected in the DNA-derived clone libraries of microorganisms of back-flushed water from the near-bottom zone of injection well 1098, sample 8 m^3 (December 2006).

Superkingdom, phylogenetic group	Representative clone of the phylotype	GenBank accession number	Number of related clones	Closest cultivable relative or clone, acc. no.	Sequence identity, %	Temperature minimum-optimum-maximum, °C
Archaea						
**DNA-based clone library of 16S rRNA genes (primers A109F and A1041r, DA library)**						
Methanosarcinales	da-93	KY273934	44	*Methanomethylovorans thermophila* L2FAW^T, AY672821	99	42–50–58
	da-38	KY273933	7	*Methanothrix soehngenii* (=”*Methanosaeta thermophila*“ DSM 4774T, LN868388)	99	Nd* –55(60)–70
Methanomicrobiales	da-84	KY273930	36	*Methanoculleus receptaculi* ZC-2^T, DQ787476	99	30–50(55)–65
	da-66	KY273932	16	*Methanolinea tarda* NOBI-1^T, AB162774	99	35–50–55
	da-37	KY273929	7	*Methanocalculus pumilus* MHT-1^T, AB008853	100	25–35–45
	da-2	KY273931	1	*Methanoregula formicica* SMSP^T, AB479390	97	10–30(33)–40
Methanococcales	da-70	KY273937	1	*Methanococcus maripaludis* C7, CP000745	99	18–38–47
Methanomassiliicoccales	da-101	KY273935	1	*Methanomassiliicoccus luminyensis* B10^T, NZ_CAJE01000013	92	25–37–45
Thermococcales	da-69	KY273936	1	*Thermococcus litoralis* DSM 5474, AY099180	99	55–85–100
Crenarchaeota	da-109	KY273938	1	Uncultured archaeon clone GR-WP33-A11, AJ583414	98	
The number of clones in the library			115			
Bacteria						
**DNA-based clone library of 16S rRNA genes 1098-8м^3-(1) (primers 8-27f and 519r, DB library)**						
Bacilli	db5-70	KY273989	100	*Geobacillus subterraneus* 34^T, AF276306	99	45–60–65
Clostridia	db5-51	KY273991	1	*Thermaerobacter nagasakiensis* JCM 11223, AB061441	99	52 – 70 –78
	db5-61	KY273990	2	Uncultured bacterium clone OF-140, KP109903	99	

Tissierellia	db5-12	KY273992	1	*Soehngenia saccharolytica* BOR-Y^T, AY353956	99	15 – 30(37) – 40
γ-Proteobacteria	db1-9	KY273974	1	*Cedecea davisae* NBRC 105702^T, AB682275	99	15 – 35–37
	db1-89	KY273975	9	*Pseudomonas poae* RE*1-1-14, NR_102514	99	
	db5-48	KY273973	1	*Thiofaba tepidiphila* DSM 19618, AB304258	96	20–45–51
β-Proteobacteria	db5-113	KY273996	1	*Tepidimonas fonticaldi* AT-A2^T, JN713899	99	35 – 55 – 60
	db1-5	KY273972	3	*Hydrogenophilus thermoluteolus* strain NBRC 14978, NR_113716	99	ND – 52 – ND
	db1-2	KY273969	1	*Thauera aminoaromatica* S2, NZ_AMXD01000247	93	4 – 28 – 40
	db5-76	KY273971	1	*Georgfuchsia toluolica* strain G5G6, NR_115995	93	
	db5-103	KY273970	1	*Azospira* sp. AK33, HF562218	99	
δ-Proteobacteria	db1-95	KY273978	2	*Syntrophobacter fumaroxidans* MPOB^T, NC_008554	92	20 – 37 – 40
	db1-3	KY273976	1	*Desulfurivibrio alkaliphilus* AHT2^T, EF422413	93	Nd – 30 –42
	db5-24	KY273977	1	*Desulfomicrobium thermophilum* P6.2, AY464939	94	37–55–60
	db1-47	KY273997	1	*Geoalkalibacter ferrihydriticus* DSM 17813^T, NR_043709	95	18 – 35 –40
Thermotogae	db1-36	KY273979	4	*Thermosipho geolei* strain DSM 13256, NR_025389	99	
	db5-82	KY273980	3	*Fervidobacterium pennivorans* DSM 9078^T, NC_017095	98	50–70–80
	db1-88	KY273981	3	*Mesotoga prima* DSM 24739 (T), NC_017934	99	20–37–50
Deferribacteres	db5-80	KY273994	3	Uncultured bacterium clone Z-47, FJ901100	98	
Chloroflexi	db1-118	KY273985	3	*Bellilinea caldifistulae* GOMI-1^T, AB243672	90	45 – 55 – 65
Nitrospira	db5-94	KY273983	1	*Thermodesulfovibrio hydrogeniphilus* Hbr5, EF081294	95	50–75–80
Actinobacteria	db5-56	KY273993	2	Uncultured *Coriobacteriales* bacterium clone OF-143, KP109904	99	
Bacteroidetes	db5-72	KY273984	1	Unidentified bacterium, strain OF1, EF148839	99	

Elusimicrobia	db1-11	KY273982	1	Uncultured *Elusimicrobium* sp. clone B30_116, KP258939	99	
Uncultured bacteria	db5-75	KY273986	1	Uncultured OD1 bacterium clone SRC61, AY193293	96	
	db1-45	KY273995	1	Candidate division JS1 bacterium enrichment culture clone B4118, HQ133192	99	
	db5-22	KY273987	1	Uncultured bacterium OP11 clone D004011B03, EU721757	98	
	db5-25	KY273988	2	Uncultured bacterium clone PNG_Kap3_B354, JF935186	98	
The number of clones in the library			153			
**DNA-based clone library of 16S rRNA genes 1098-8m^3-(2) (primers Pla46f and 519r, DP library)**						
Candidate division *Cloacimonetes* (WWE1)	dp-92	KY273999	57	Candidatus *Cloacamonas acidaminovorans*, CU466930	99	
	dp-88	KY273998	26	Uncultured WWE1 bacterium clone QEDQ2AE08, CU923265	98	
Planctomycetes	dp-8	KY274000	3	*Thermogutta terrifontis* R1^T, KC867694	90	25 – 55/67 – 67
Lentisphaerae	dp-97	KY274001	3	*Victivallis vadensis* ATCC BAA-548^T, AY049713	84	20 – 37 – 40
The number of clones in the library			89			
**DNA-based clone library of *alkB* genes (primers Alk-BFB and Alk-BRB)**						
	k8-10	KY273967	22	*Geobacillus* sp. *alkB-geo1*, EF534176	99	
	k8-16	KY273968	5	*Geobacillus* sp. *alkB-geo6*, EF534175	98	
	k8-2	KY273966	16	Uncultured bacterium clone P3E03_GMR	98	
The number of clones in the library			43			

***** No data**.**

Supplementary Table S3. Phylogenetic diversity of cDNA of 16S rRNA sequences of *Bacteria* detected in the RNA-derived clone library of microorganisms of back-flushed water from the near-bottom zone of injection well 1098, sample 8 m^3 (RB library) (December 2006).

Phylogenetic group	Representative clone of the phylotype	GenBank accession number	Number of related clones	Closest cultivable relative or clone, acc. no.	Sequence identity, %	Temperature minimum-optimum-maximum, °C
β-Proteobacteria	r8-221	KY273943	36	*Tepidimonas fonticaldi* AT-A2^T, JN713899	100	35 – 55 – 60
	r8-191	KY273941	44	*Thauera aminoaromatica* S2^T, AMXD01000247	92	4 – 28 – 40
	r8-125	KY273940	5	*Azoarcus tolulyticus* 2FB6, AF229861	95	25–ND--37
	r8-28	KY273939	2	*Denitromonas indolicum* strain MPKc, AY972852	94	
	r8-12	KY273942	2	*Tepidiphilus margaritifer* strain N2-214, NR_025556	99	25 – 50 – 61
δ-Proteobacteria	r8-210	KY273951	5	*Desulfomicrobium thermophilum* P6.2^T, AY464939	99	37 – 55 – 60
	r8-264	KY273949	3	*Geobacter hydrogenophilus* H2^T, U28173	97	ND – 35 – ND
	r8-254	KY273948	2	*Desulfuromonas michiganensis* BB1^T, AF357915	96	10 – 25(30) – ND
	r8-140	KY273947	10	Uncultured bacterium clone Phox-21, KU898264	99	
	r8-265	KY273950	1	*Desulfurivibrio alkaliphilus* AHT2^T, NC_014216	92	Nd – 30 – 42
	r8-35	KY273953	1	*Desulfatitalea tepidiphila* S28bF^T, AB614135	90	13 – 34(42) – 45
	r8-153	KY273952	1	*Smithella propionica* LYP^T, AF126282	88	25 – 35(37) – 37
γ-Proteobacteria	r8-4	KY273944	4	*Thiofaba tepidiphila* BDA453^T, AB304258	99	20 – 45 – 51
	r8-216	KY273946	2	*Pseudomonas poae* RE*1-1-14, NR_102514	99	5 – 25(30) – 37
	r8-151	KY273945	1	*Acinetobacter johnsonii* A13, KT767653	99	15 – 30 – 37
Deferribacteres	r8-26	KY273964	10	*Flexistipes* sp. vp.180, AF220344	91	30 – 46 – 53
	r8-229	KY273963	6	*Calditerrivibrio nitroreducens* DSM 19672^T, NC_014758	99	30 – 55 – 65
Bacteroidetes	r8-134	KY273957	6	*Fluviicola taffensis* DSM 16823^T, CP002542	86	
	r8-204	KY273958	1	Unidentified bacterium, strain OF1, EF148839	99	
Chloroflexi	r8-238	KY273959	2	*Bellilinea caldifistulae* GOMI-1, AB243672	89	
	r8-241	KY273960	1	*Bellilinea caldifistulae* GOMI-1, AB243672	99	45 – 55 – 65
Ignavibacteriae	r8-248	KY273956	1	Uncultured bacterium clone TTMF57, AY741701	96	
	r8-5	KY273955	1	*Melioribacter roseus* strain P3M-2, NR_074796	99	37– 47 – 60

Elusimicrobia	r8-133	KY273954	1	Uncultured *Elusimicrobium* sp. clone B30_116, KP258939	99	
Uncultured bacteria	r8-91	KY273965	1	Uncultured bacterium clone ARWH-BH02, AB546037	99	
	r8-168	KY273962	1	Uncultured bacterium clone OTU-R02, FJ164077	99	
	r8-57	KY273961	1	Bacterium enrichment culture clone R4-11B, GU196193	97	
The number of clones in the library			151			

Supplementary figures

Supplementary Figure 1.

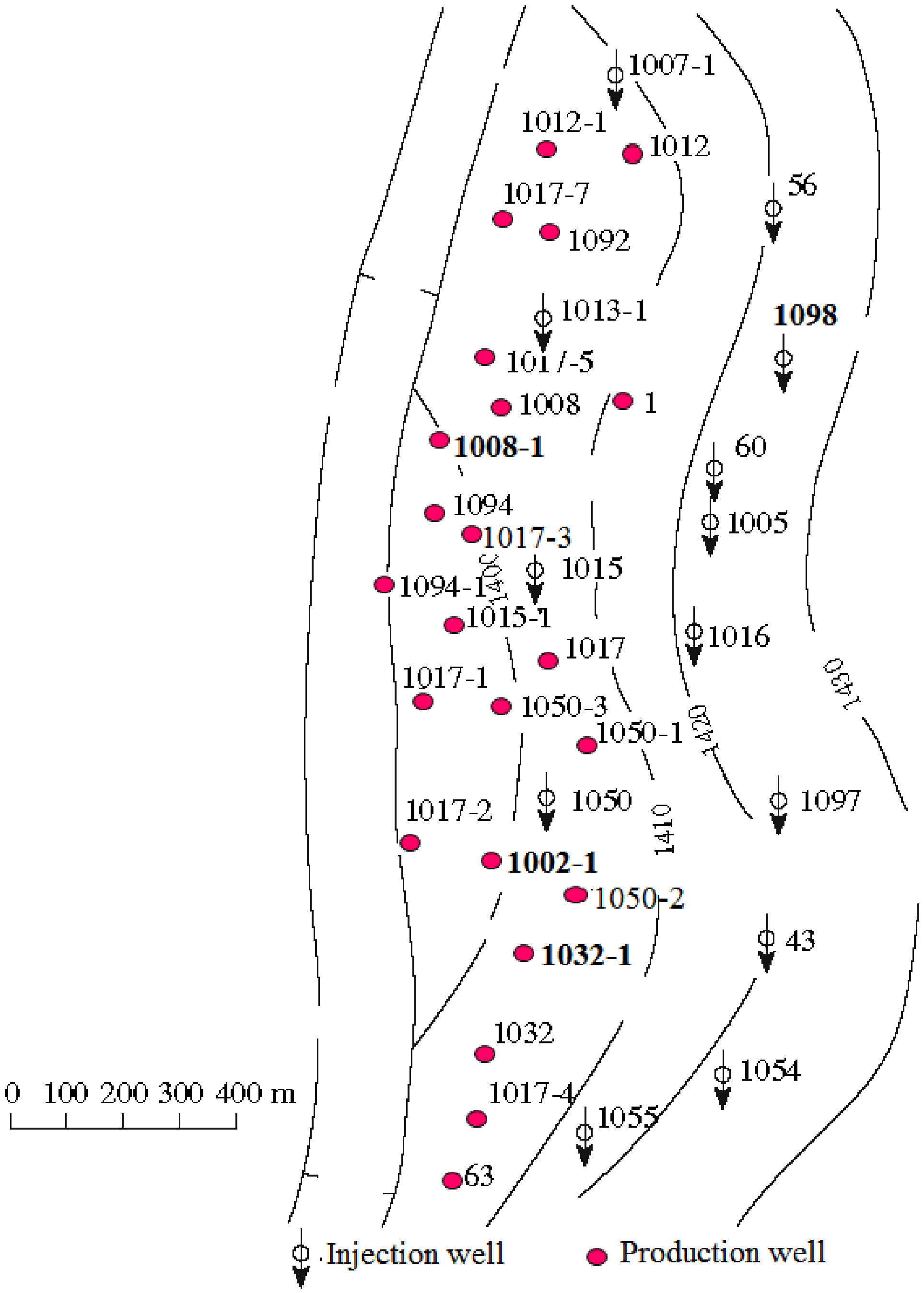

Supplementary Figure S1. Layout of the injection and production wells at the trial site of the Kongdian bed. The numbers of injection well 1098 and production wells 1002-1, 1008-1 and 1032-1 studied in this paper are in bold.

Supplementary Figure S2.

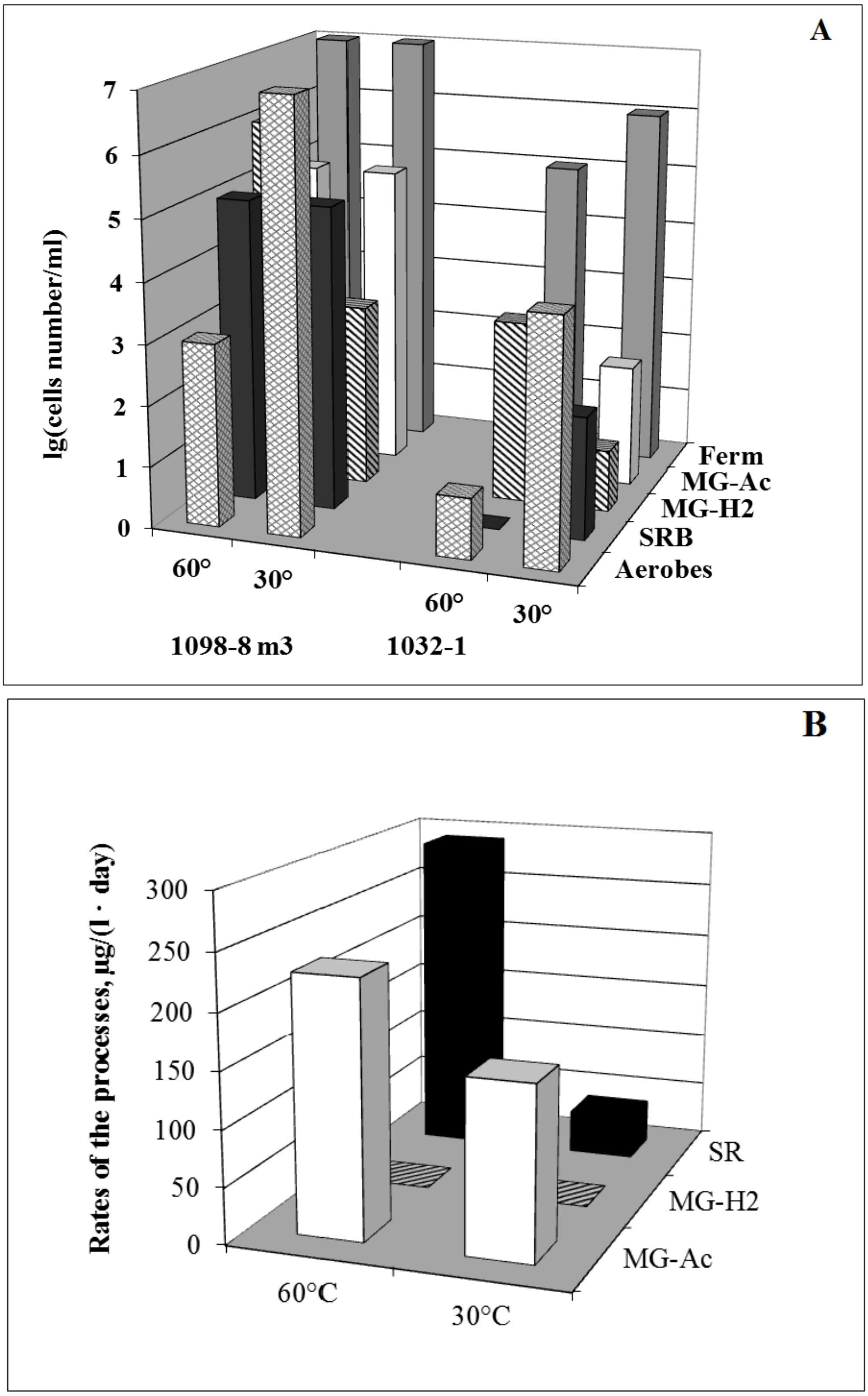

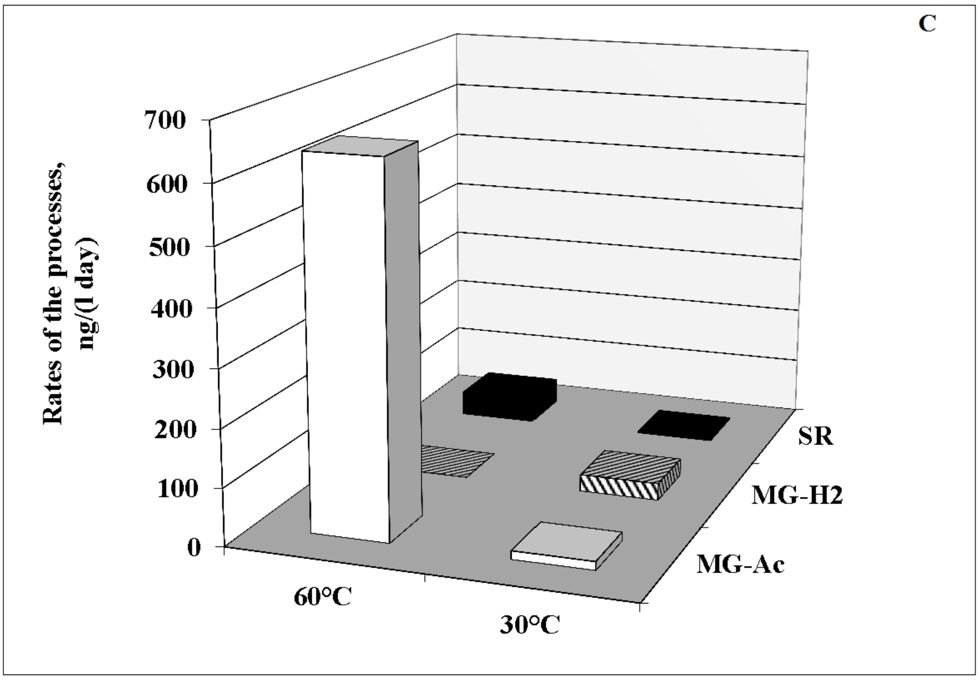

Supplementary Figure S2. Abundance of thermophilic and mesophilic aerobic organotrophs (Aerobes) and anaerobic fermenting (Ferm) and sulfate-reducing (SRB) bacteria and methanogens [in the medium with H_2+CO_2 (MG-H2) and acetate (MG-Ac)] (A) and the rates of thermophilic and mesophilic sulfate reduction (SR) and methanogenesis from 2-^{14}C-acetate (MG-Ac) and from NaH^{14}CO$_3$ (MG-H2) in the water from the near-bottom zone of injection well 1098 (8 m^3) (B) and from production well 1032-1 (C) (June 2002).

Supplementary Figure S3.

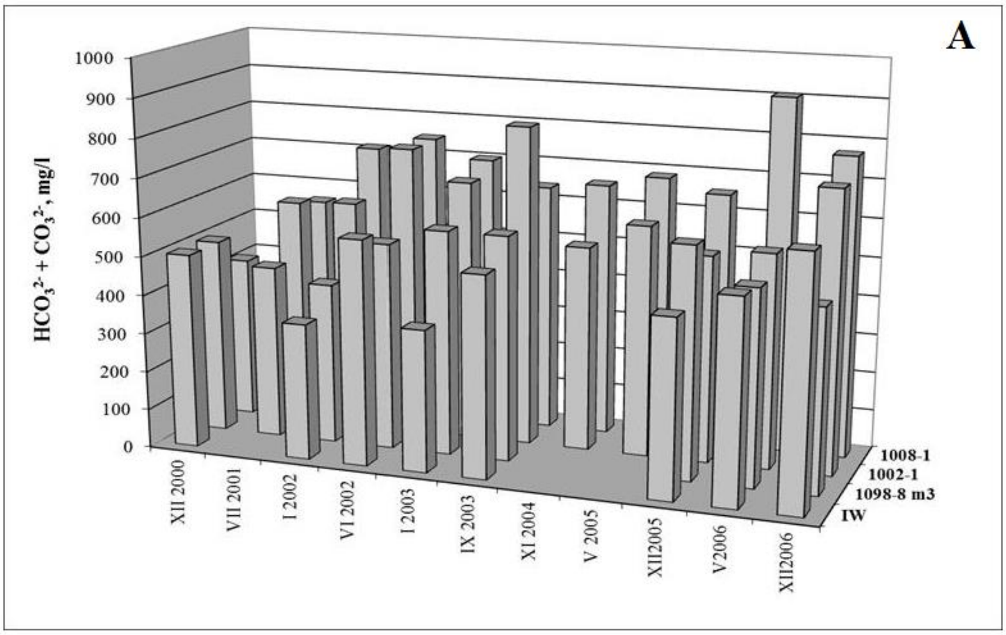

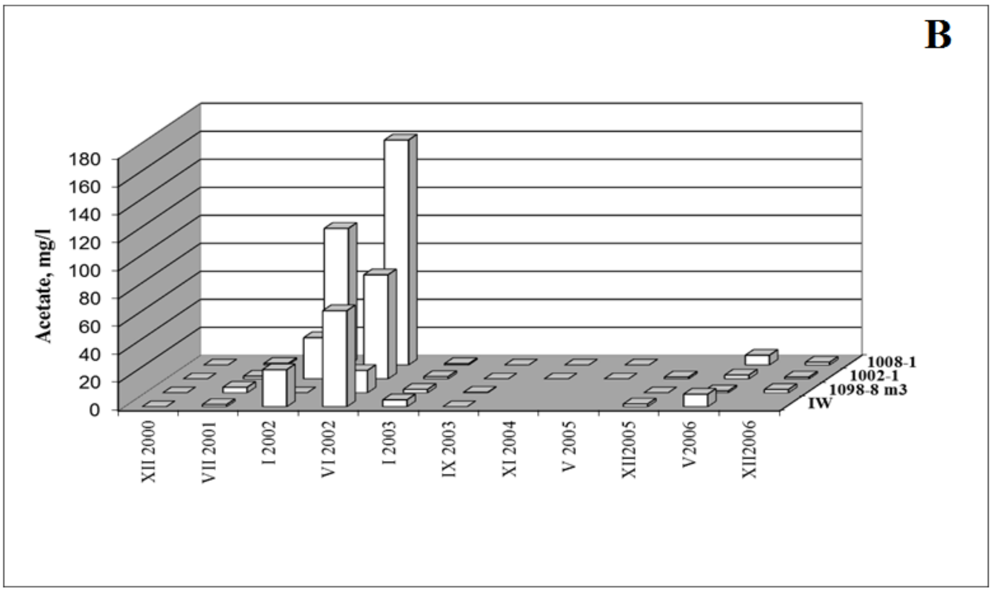

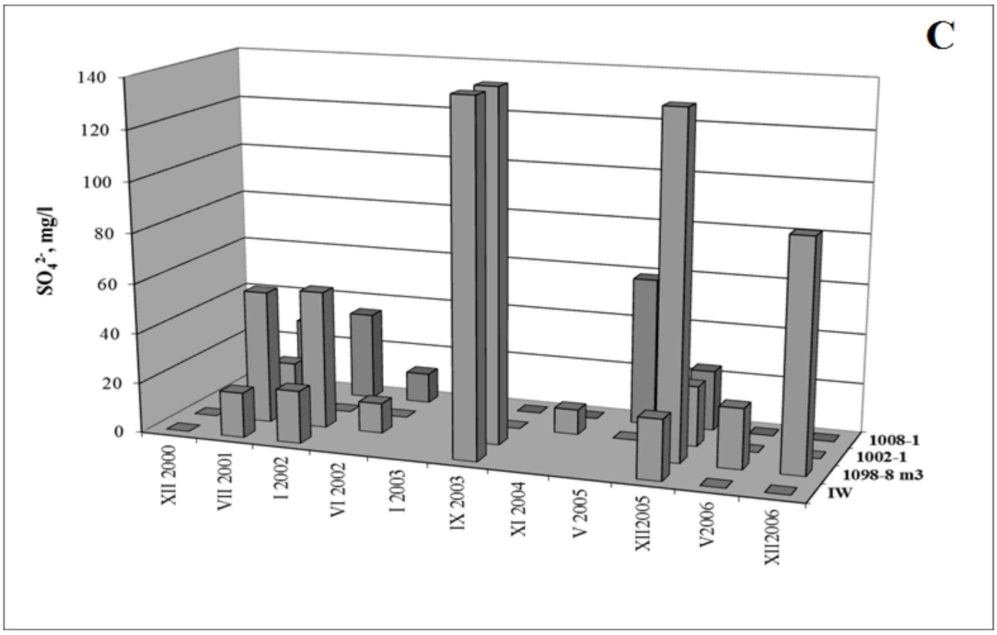

Supplementary Figure S3. Concentrations of mineral carbonates (HCO_3^- + CO_3^{2-}) (A), acetate (B), and sulfate (C) in injection water (IW), in back-flushed water from near-bottom zone of injection well (1098-8 m3) and in water from production wells 1002-1 and 1008-1 of the Kongdian bed during the biotechnology trial.

Supplementary Figure S4.

(A)

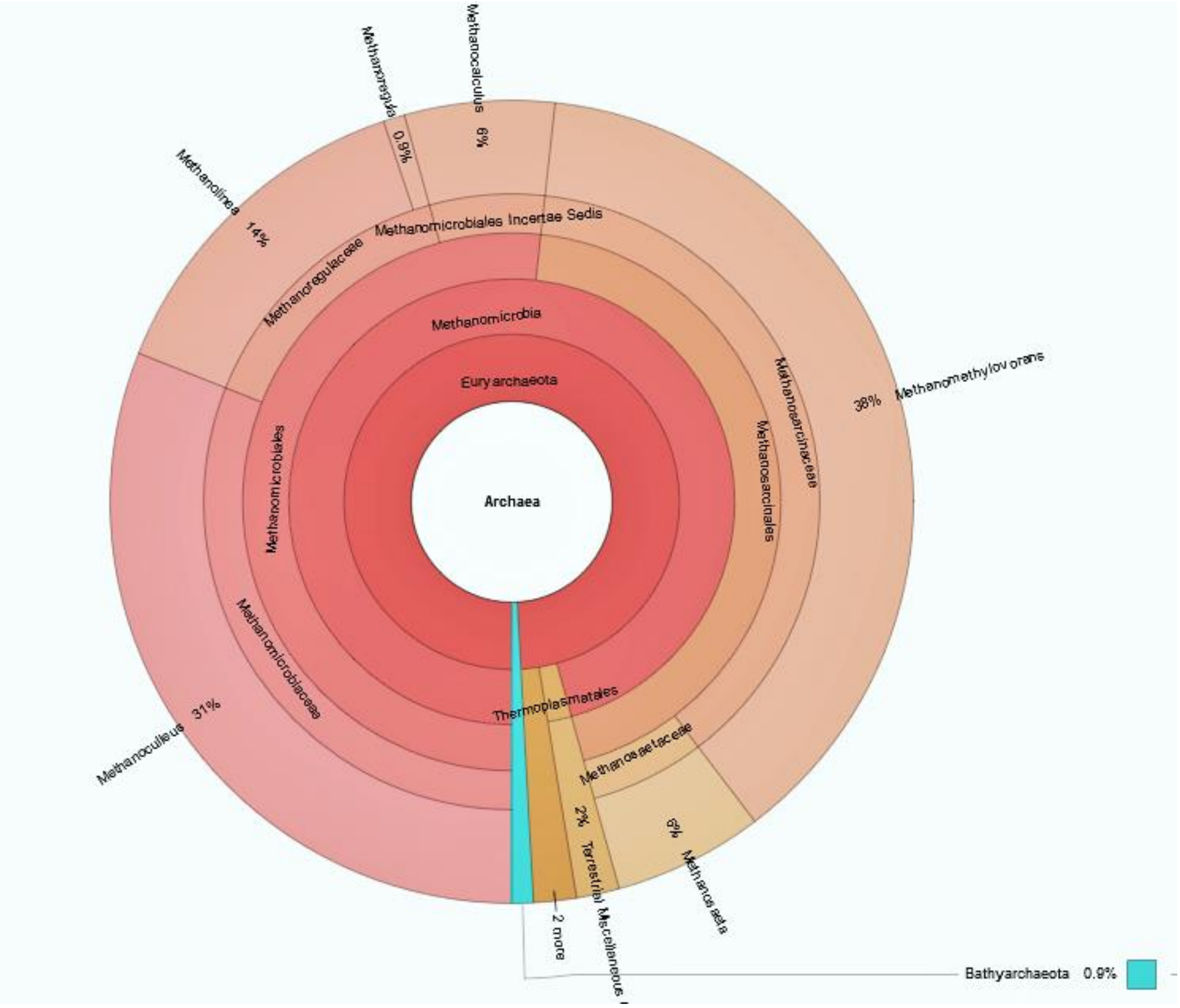

(B)

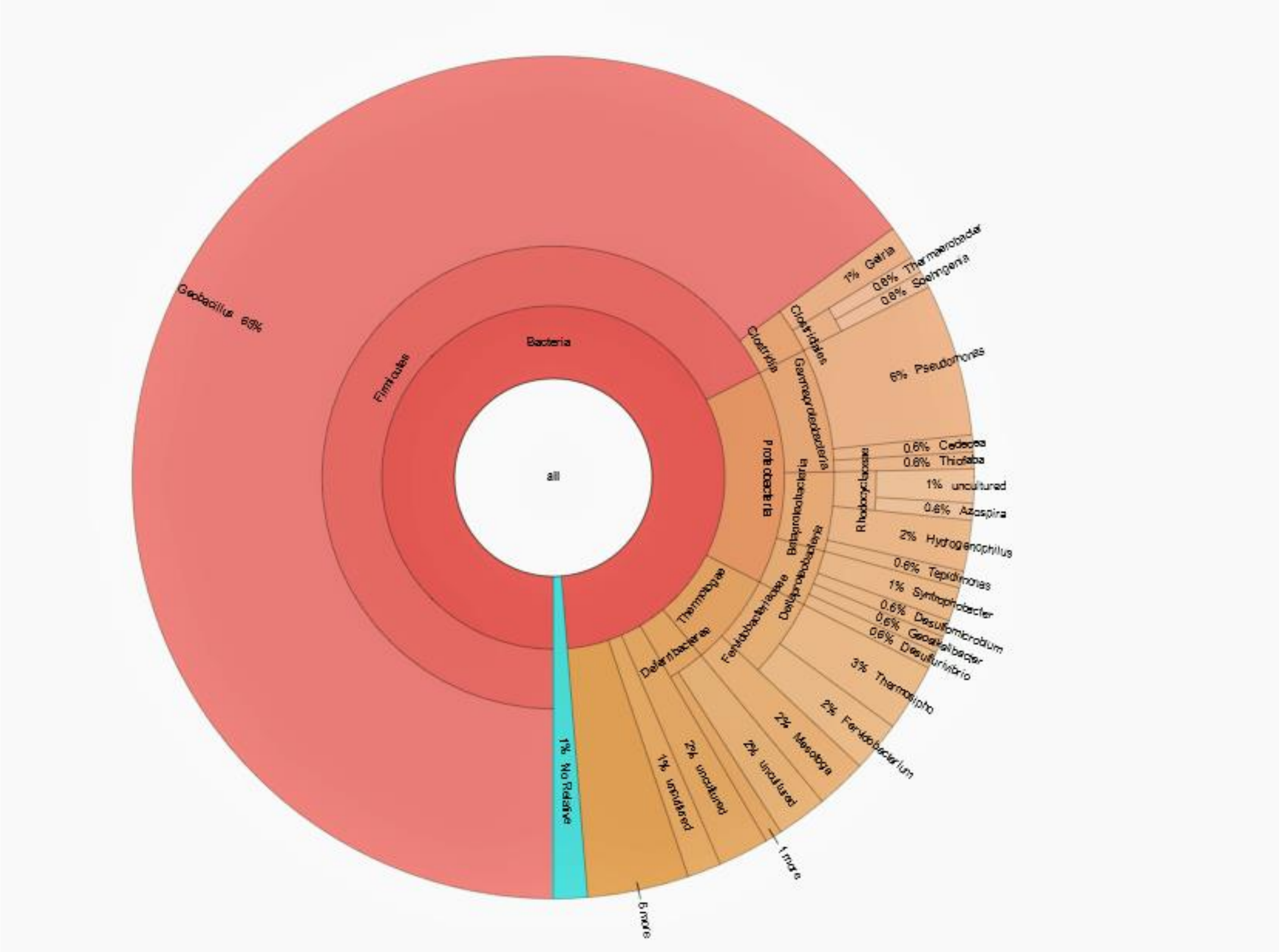

all
Bacteria
Firmicutes
Geobacillus 66%
Proteobacteria
Clostridia
Clostridiales
1% Gelria
0.6% Thermaerobacter
0.6% Spelngenia
Gammaproteobacteria
6% Pseudomonas
0.6% Cedecea
0.6% Thiofaba
Betaproteobacteria
Rhodocyclaceae
1% uncultured
0.6% Azospira
2% Hydrogenophilus
0.6% Tepidimonas
Deltaproteobacteria
1% Syntrophobacter
0.6% Desulfomicrobium
0.6% Geoalkalibacter
0.6% Desulfurivibrio
Thermotogae
Fervidobacteriaceae
3% Thermosipho
2% Fervidobacterium
2% Mesotoga
2% uncultured
Deferribacteres
2% uncultured
1% uncultured
1% No Relative
1 more
6 more

(C)

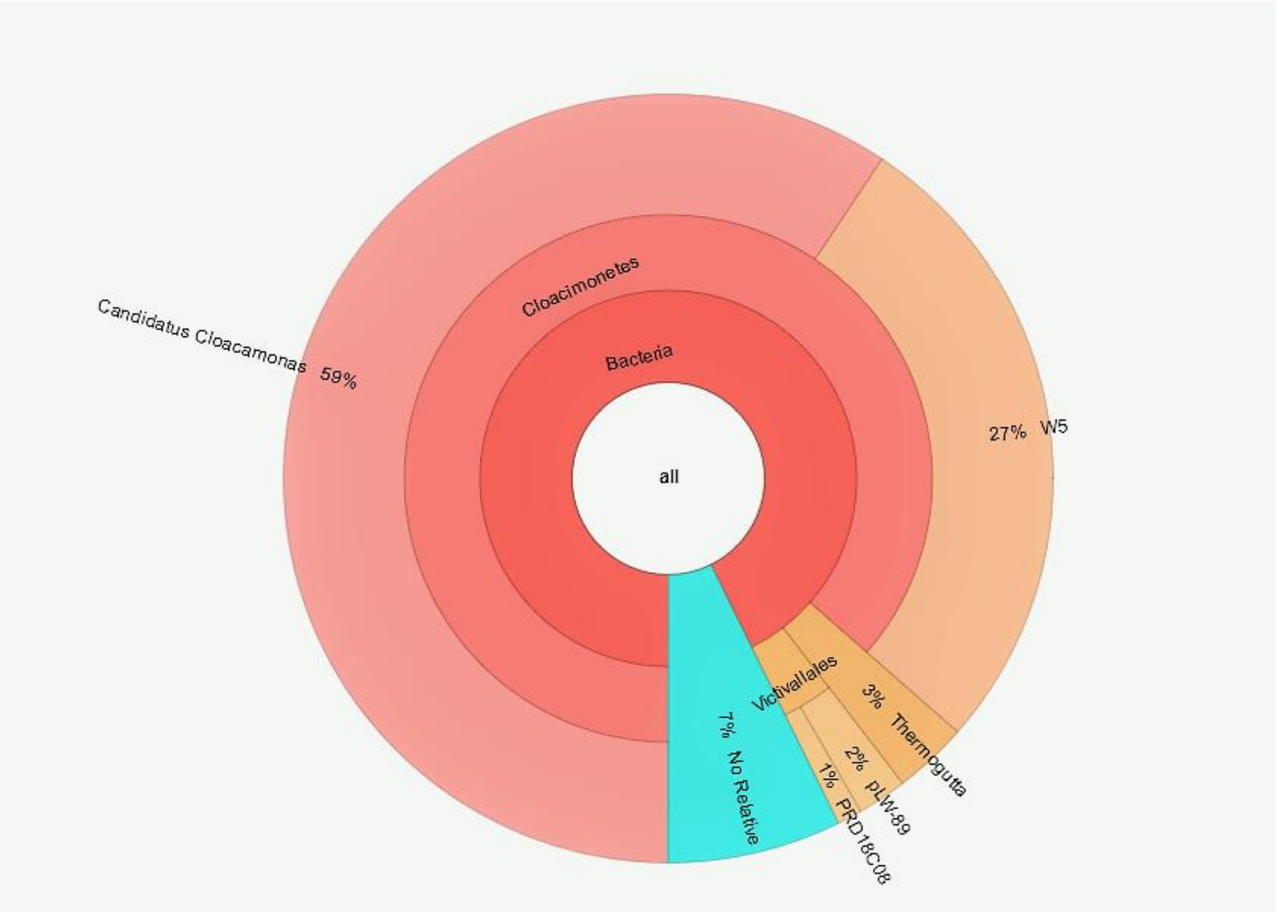

all
Bacteria
Cloacimonetes
Candidatus Cloacamonas 59%
27% W5
7% No Relative
Victivallales
3% Thermogutta
2% pLW-89
1% PRD18C08

(D)

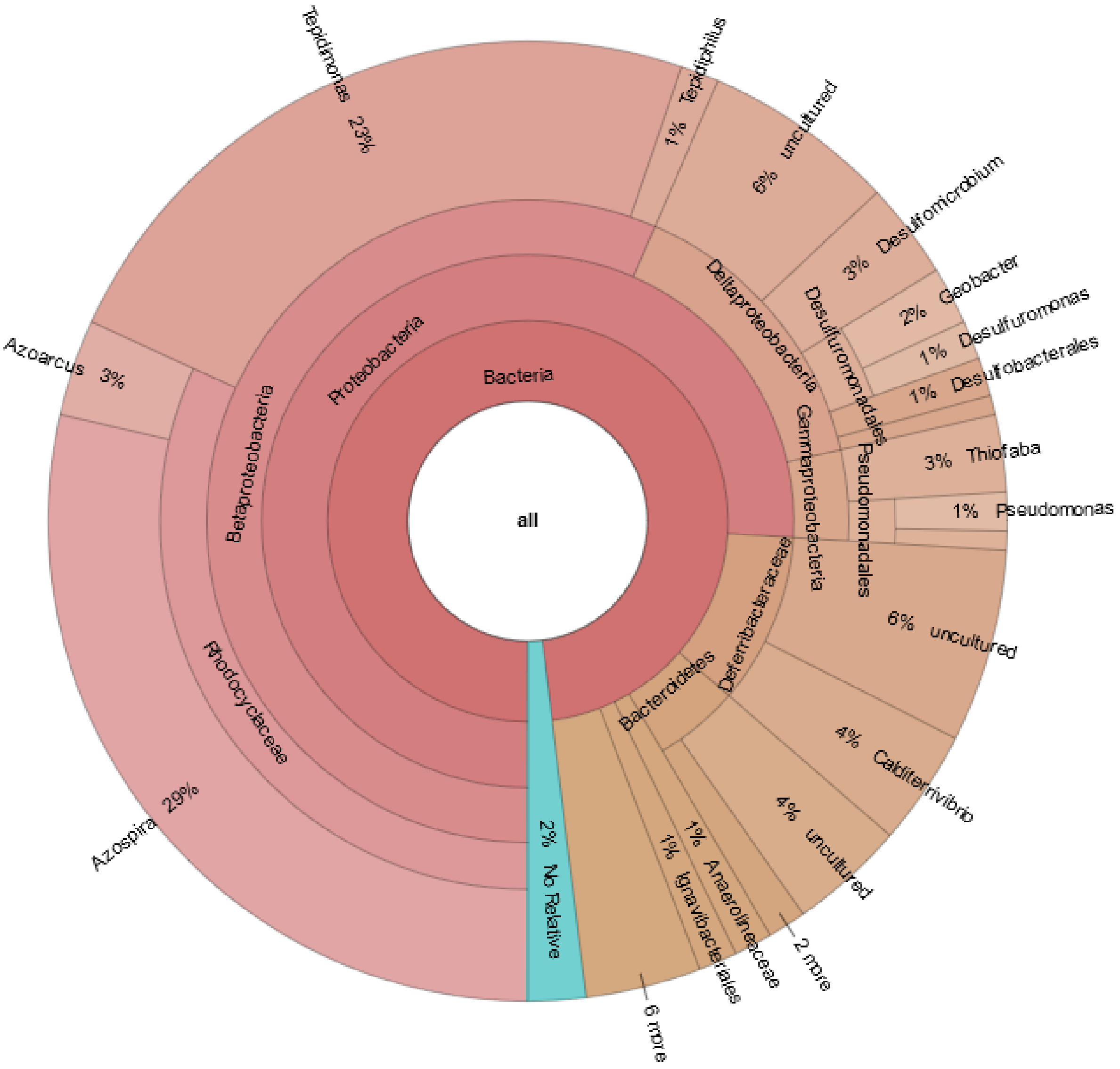

all
Bacteria
Proteobacteria
Betaproteobacteria
Rhodocyclaceae
Tepidimonas 23%
1% Tepidiphilus
6% uncultured
Deltaproteobacteria
Desulfuromonadales
3% Desulfomicrobium
2% Geobacter
1% Desulfuromonas
1% Desulfobacterales
Gammaproteobacteria
Pseudomonadales
3% Thiofaba
1% Pseudomonas
Azoarcus 3%
Azospira 29%
Deferribacteraceae
6% uncultured
4% Calditerrivibrio
4% uncultured
Bacteroidetes
1% Anaerolineaceae
1% Ignavibacteriales
2 more
6 more
2% No Relative

(E)

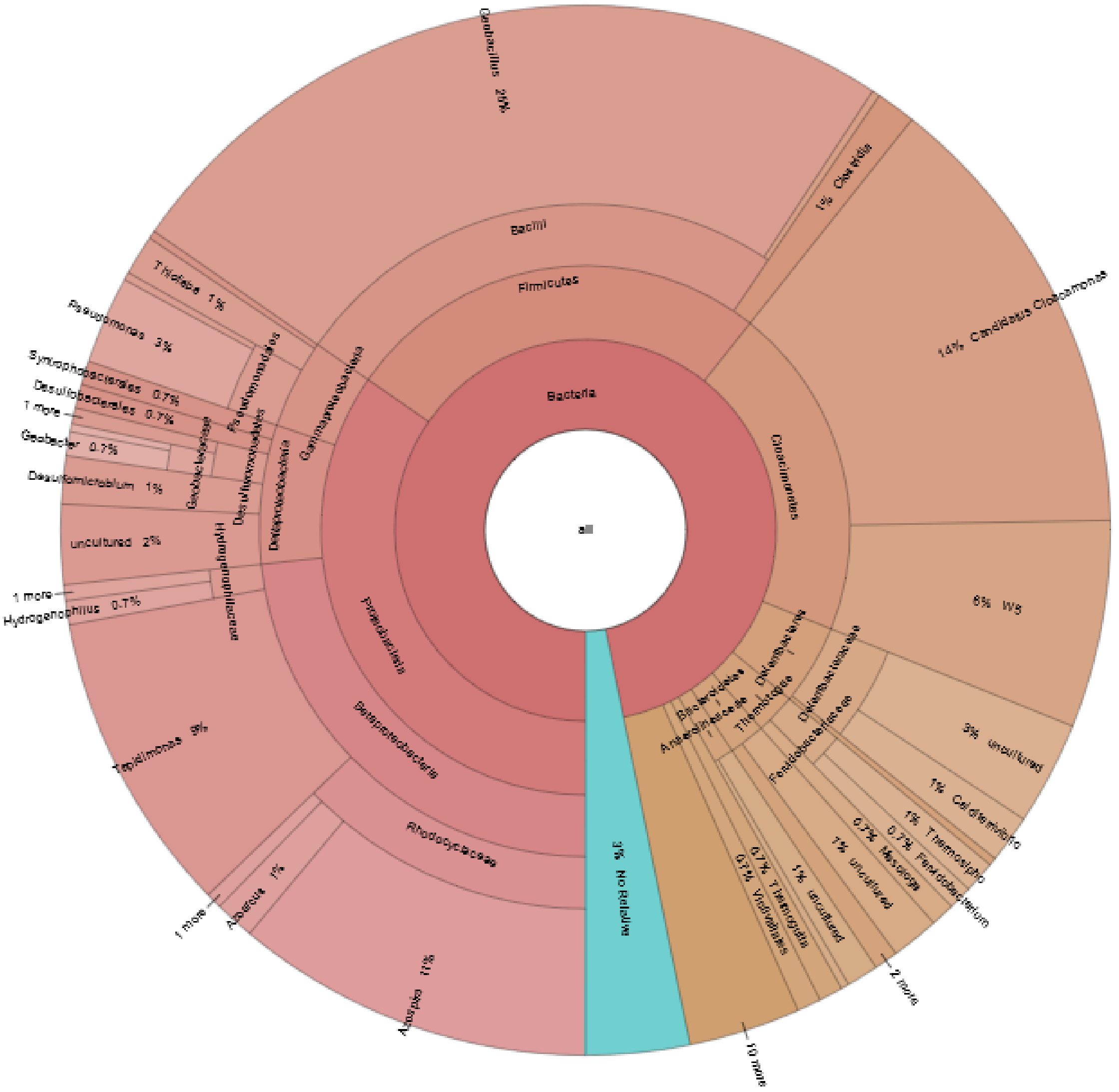

Supplementary Figure S4. Diversity of 16S rRNA genes of *Archaea* (DA library, A) and *Bacteria* (DB (B) and DP (C) libraries) in DNA-derived libraries; diversity of cDNA of 16S rRNA sequences of *Bacteria* in RNA-derived library RB (D); and total bacterial diversity in DB, DP and RB libraries (E). Analyses were performed on the base of SILVAngs pipeline (December 2006).
